# Optimized Ebselen-Based Inhibitors of Bacterial Ureases with Nontypical Mode of Action

**DOI:** 10.1021/acs.jmedchem.2c01799

**Published:** 2023-01-20

**Authors:** Katarzyna Macegoniuk, Wojciech Tabor, Luca Mazzei, Michele Cianci, Mirosław Giurg, Kamila Olech, Małgorzata Burda-Grabowska, Rafał Kaleta, Agnieszka Grabowiecka, Artur Mucha, Stefano Ciurli, Łukasz Berlicki

**Affiliations:** † Department of Bioorganic Chemistry, Wrocław University of Science and Technology, Wybrzeże Wyspiańskiego 27, 50-370 Wrocław, Poland; ‡ Laboratory of Bioinorganic Chemistry, Department of Pharmacy and Biotechnology (FaBiT), University of Bologna, Viale Giuseppe Fanin 40, 40138 Bologna, Italy; § Department of Agricultural, Food and Environmental Sciences, Polytechnic University of Marche, Via Brecce Bianche 10, 60131 Ancona, Italy; ∥ Department of Organic and Medicinal Chemistry, Wrocław University of Science and Technology, Wybrzeże Wyspiańskiego 27, 50-370 Wrocław, Poland

## Abstract

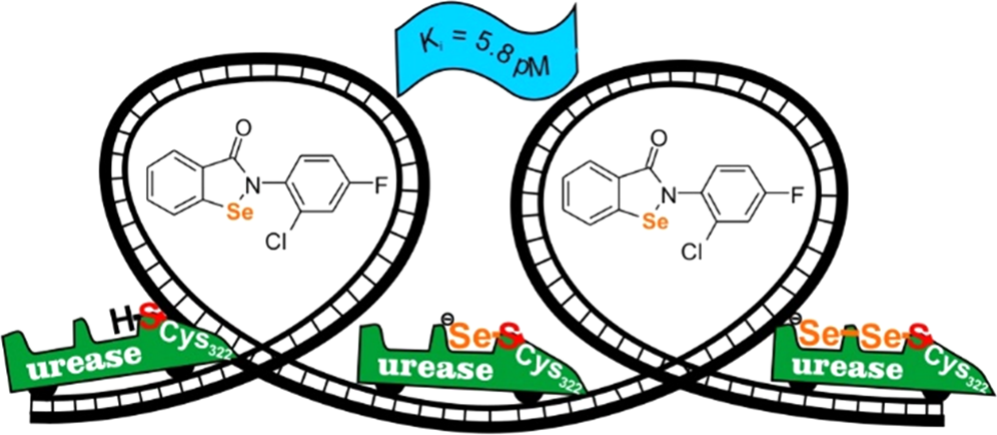

Screening of 25 analogs of Ebselen, diversified at the N-aromatic residue, led to the identification of the most potent inhibitors of *Sporosarcina pasteurii* urease reported to date. The presence of a dihalogenated phenyl ring caused exceptional activity of these 1,2-benzisoselenazol-3(2*H*)-ones, with *K_i_* value in a low picomolar range (<20 pM). The affinity was attributed to the increased π–π and π–cation interactions of the dihalogenated phenyl ring with αHis323 and αArg339 during the initial step of binding. Complementary biological studies with selected compounds on the inhibition of ureolysis in whole *Proteus mirabilis* cells showed a very good potency (IC_50_ < 25 nM in phosphate-buffered saline (PBS) buffer and IC_90_ < 50 nM in a urine model) for monosubstituted N-phenyl derivatives. The crystal structure of *S. pasteurii* urease inhibited by one of the most active analogs revealed the recurrent selenation of the Cys322 thiolate, yielding an unprecedented Cys322-S–Se–Se chemical moiety.

## Introduction

Ebselen (2-phenyl-1,2-benzisoselenazol-3(2*H*)-one, **1**; Table 1) is an extensively studied antioxidant, anti-inflammatory, antiatherosclerotic, and cytoprotective organoselenium compound.^1^ While these properties were originally attributed to a glutathione peroxidase-like activity of Ebselen,^2−4^ the biomedical impact of this drug in living organisms has been shown to be much more complex. Accordingly, Ebselen and its metabolites react with hydroperoxides to protect cells from free-radical damage;^5,6^ furthermore, Ebselen works as a substrate for thioredoxin reductase,^7^ while the inhibition at low concentrations of a number of enzymes involved in inflammation, such as lipoxygenases, NO synthases, NADPH oxidase, and others, has also been well documented and reviewed.^8−10^ In addition to the important functions associated with the redox state and antioxidant defense, Ebselen has been recently explored as a low-molecular-weight lead compound to develop efficient inhibitors of multiple enzymes of different classes and origins. These enzymes include potential targets for anticancer treatment, such as histone deacetylases (*K_i_* = 0.08–4.42 μM),^11^ methionine aminopeptidase 2 (IC_50_ = 2.43 μM),^12^ glutamate dehydrogenase (IC_50_ = 0.36 μM),^13−15^ and 6-phosphogluconate dehydrogenase (IC_50_ ∼0.07 μM).^16^ Ebselen-based pharmaceuticals are in clinical trials for the treatment of cardiovascular diseases, arthritis, and atherosclerosis, despite the evidence of cellular toxicity^9,17^ due to nonspecific thiol-oxidizing properties and inhibition of cysteine-containing proteins. However, a highly specific inactivation of thiols in enzymes is considered therapeutic significance.^1,17^

**Table 1:**
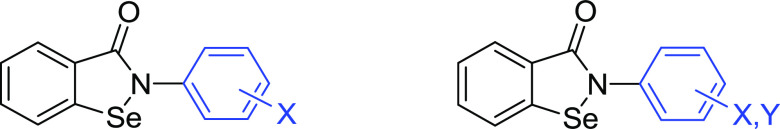
Structures and Inhibitory Activity of *N*-Phenyl-1,2-benzisoselenazol-3(2*H*)-ones Mono/Disubstituted on the Phenyl Ring (**30**–**42** and **43**–**54**, Respectively) against *Sporosarcina pasteurii* Urease (the Most Significant Inhibition Values are Indicated in Bold)

entry	X	*K_i_* [nM]	entry	X	Y	*K_i_* [nM]
**1**	H	2.11 ± 0.18^24^				
**30**	2-Me	3.56 ± 0.32	**43**	2-Me	4-OMe	7.30 ± 0.56^a^
**31**	2-F	0.974 ± 0.089	**44**	2-F	4-F	**0.0167** ± **1.7** × **10**^**–3**^
**32**	2-Cl	13.7 ± 0.96	**45**	2-F	4-Cl	**5.26** × **10**^**–3**^ ± **4.1** × **10**^**–4**^
**33**	2-Br	6.79 ± 0.53	**46**	2-Cl	4-Me	3.26 ± 0.23
**34**	2-OH	1.42 ± 0.14	**47**	2-Cl	4-F	**5.80** × **10**^**–3**^ ± **4.0** × **10**^**–4**^
**35**	2-OMe	5.36 ± 0.43	**48**	2-Me	5-Cl	75.2 ± 6.2
**36**	3-F	1.89 ± 0.16	**49**	2-F	5-Cl	**0.0135** ± **1.4** × **10**^**–3**^
**37**	3-OMe	1.07 ± 0.080^a^	**50**	2-Cl	5-Me	1.63 ± 0.13
**38**	4-Me	4.04 ± 0.32	**51**	2-Cl	5-Cl	**8.85** × **10**^**–3**^ ± **6.7** × **10**^–**4**^
**39**	4-F	24.1 ± 2.6	**52**	2-OMe	5-Me	7.11 ± 0.56^a^
**40**	4-CF_3_	**0.0363** ± **3.9** × **10**^**–3**^	**53**	2-OMe	5-Cl	1.63 ± 0.12
**41**	4-OMe	44.0 ± 4.2	**54**	3-Me	4-Cl	2.45 ± 0.16
**42**	4-O-*n*-Bu	1.78 ± 0.18^a^				

a Slow-binding kinetics (for more details, see the Supporting Information).

The antibacterial properties of Ebselen are also ascribed to its multifaceted reactivity with enzymes and protein thiols. Rational design, repurposing, and high-throughput screening studies have validated multiple microbial molecular targets for organoselenium compounds. To mention representative examples, these microbial targets are involved in the survival and development of β-lactam-resistant strains that produce New Delhi metallo-β-lactamase-1 (*K_i_* = 0.38 μM)^18^ and those responsible for multidrug-resistant *Staphylococcus aureus* infections (MIC ranging from 0.125 to 0.5 μg/mL);^19^ additional targets are the antigen 85 complex required for the biosynthesis of the *Mycobacterium tuberculosis* cell wall (*K_i_* = 0.063 μM)^20,21^ and *Clostridium difficile* major virulence factor toxin B (IC_50_ = 6.9 nM).^22^ Most of all, Ebselen and its analogs have been shown to act as inhibitors of bacterial thioredoxin reductase, for example, *Escherichia coli* (*K_i_* = 0.52 μM)^23^ or *Bacillus anthracis* (IC_50_ = 1.0 μM),^24^ and exhibit potent antimicrobial activity against a range of Gram-positive species, such as *Bacillus subtilis*, *S. aureus*, *Bacillus cereus*, and *M. tuberculosis*.

Organoselenium compounds, and, in particular, Ebselen, have been classified among the most potent low-molecular-weight inhibitors of bacterial ureases.^25^ Urease, depending on the organism, is a nickel-containing homo- or heterooligomeric amidohydrolase that is commonly expressed in plants, fungi, and bacteria, but not in animals/humans, and catalyzes the decomposition of urea to ammonia and carbonate.^26−28^ The activity of urease in microorganisms, which in turn determines the accumulation of NH_3_ and the increase in pH in the microbial microenvironment, is a key factor contributing to the persistence of notorious bacterial infections. Consequently, *Helicobacter pylori*, a Gram-negative bacterium that can survive in the acidic stomach environment, induces gastric inflammation and increases the risk of developing duodenal and gastric ulcers, adenocarcinoma, and lymphoma.^26,29,30^ In addition to gastrointestinal infections, problems in human health caused by ureolytic bacteria, in particular, *Proteus mirabilis* and *Staphylococcus saprophyticus*, concern the urinary tract, wounds, and bloodstream infections, which are mostly acquired upon hospitalization. The increased pH of the urinary tract mediated by *P. mirabilis* facilitates the formation of crystals of carbonate apatite and struvite.^30,31^ Crystalline biofilms formed specifically by *P. mirabilis* on the inner surface of catheters and urothelium are responsible for the decrease in susceptibility to treatment agents and the notable recurrence of infections. The low-permeability asymmetrical outer membrane rich in efflux pumps gives this microorganism the intrinsic ability to regulate antibiotic influx, which is further enhanced by the growing evolution of antibiotic-inactivating enzymes.^32^ Interestingly, the invariantly high susceptibility of *P. mirabilis* to ciprofloxacin (fluoroquinolone, which affects cell division) has been attributed to the inhibition of ureases, in addition to the main biological activity of this compound.^33,34^

A series of 1,2-benzisoselenazol-3(2*H*)-ones and their open-cycle diselenide derivatives have recently been investigated as urease inhibitors. Ebselen was found to inactivate *S. pasteurii* and *H. pylori* ureases with *K_i_* in the nanomolar range,^25^ and it was suggested that the inhibitor acted covalently and irreversibly, similar to what was previously evidenced and reported for other proteins.^35^ Although Ebselen is considered to act as a broad-band thiol-targeted inhibitor, it inactivated *S. pasteurii* urease (SPU) with a *K_i_* of 2.11 nM, an exceptional value among mammalian or bacterial enzymes.

In the present study, further improvements in the potency of Ebselen as a urease inhibitor have been achieved: testing dedicated structural modifications of the basic compound skeleton led to the identification of Ebselen derivatives that act as *picomolar* inhibitors of SPU. Crystallographic studies on SPU inhibited by the most efficient Ebselen derivative reveal the molecular basis for this striking reactivity, namely, the formation of a unique chemical modification of the catalytic cysteine thiol, a novel mechanism of urease inhibition.

## Results and Discussion

As previously reported, 2-phenyl-1,2-benzisoselenazol-3(2*H*)-one (Ebselen, **1**; Table 1) inhibited SPU with *K_i_* = 2.11 nM.^25^ These observations suggested a mechanism of action of these compounds that involved the reaction with a conserved cysteine residue (αCys322 for SPU), located at the entrance of the active site and critical to catalysis.^28,36−42^ The reaction resulted in the formation of a covalent S–Se adduct between the catalytic cysteine thiol and the selenium atom of Ebselen.^25^ However, no hard proof for this mechanism has been provided. In addition, the organoselenium compounds confirmed their cell membrane-penetrating capability and antiureolytic activity in whole cells of *E. coli* and *H. pylori* models, *in vitro*.

**Scheme 1:**
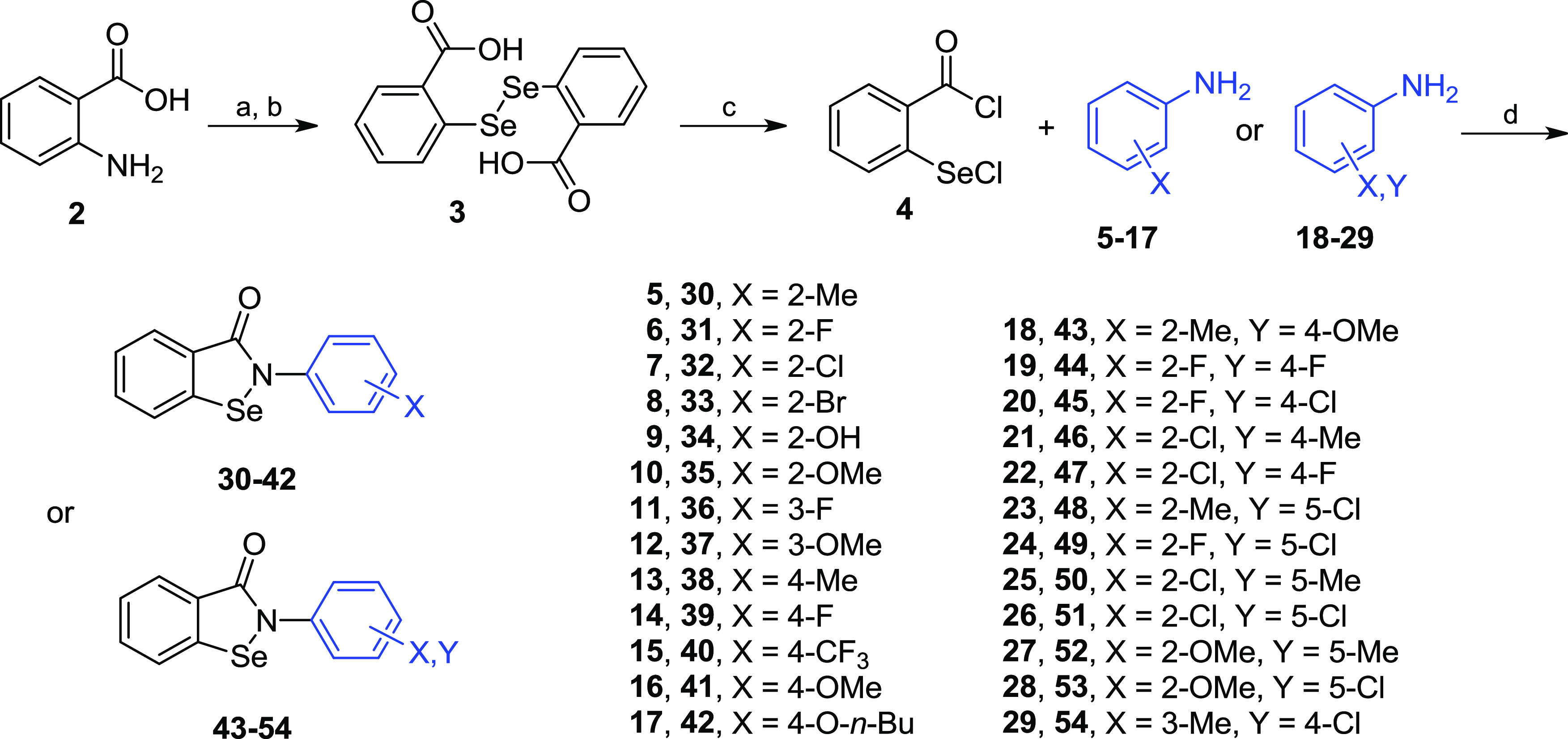
Reagents and Conditions: (a) NaNO_2_, HCl, 0–5 °C; (b) Li_2_Se_2_, NaOH, −7–0 °C, then HCl; (c) SOCl_2_ (excess), DMF (cat.), Δ; and (d) MeCN or CH_2_Cl_2_, Et_3_N

The present study aimed to expand the scope of the structural diversity of selenium-based inhibitors and to verify the influence of certain modifications on the potency against bacterial ureases. To achieve this goal, a selection of substituents on the phenyl ring of Ebselen, with both structural and functional effects, was chosen. These Ebselen derivatives consisted of monosubstitution with alkyl, hydroxyl, and alkoxy groups, as well as different halogen atoms, located in the *ortho*, *meta*, or *para* positions (**30**–**42**, Scheme 1 and Table 1); additional disubstituted regioisomers were also included (**43**–**54**, Scheme 1 and Table 1). Compounds were synthesized according to well-established procedures.^12,43−45^ Thus, 2-benzisoselenazol-3(2*H*)-ones **30**–**54** were obtained by the reaction of anilines **5**–**29** with 2-(chloroseleno)benzoyl chloride (**4**, Scheme 1). Chloride **4** was available by the chlorination of bis(carboxyphenyl)diselenide **3** with thionyl chloride, while diacid **3** was synthesized by diazotization of anthranilic acid **2** followed by the reaction with dilithium diselenide.^44^ The set of 25 derivatives of Ebselen was then characterized with respect to their potency in inhibiting bacterial urease (Table 1).

The inhibitory activity of 2-aryl-1,2-benzisoselenazol-3(2*H*)-ones against SPU varied from significant to exceptional. Among monosubstituted analogs (Table 1, left column, **30**–**42**), the modification of the phenyl ring of Ebselen at positions 2 (*ortho*) and 3 (*meta*) showed marginal effects on activity compared to lead compound **1** as it remained at a low nanomolar level (*K*_i_ = 0.974–13.7 nM for **30**–**37** vs *K_i_* = 2.11 nM for **1**). Specifically, *ortho*-F (**31**), *ortho*-OH (**34**), *meta*-F (**36**), and *meta*-OMe (**37**) induced a slight improvement of inhibitory constants (*K_i_* = 0.974–1.89 nM). The potency of the *para* isomers (**38**–**42**) was more dispersed. In particular, the *para*-trifluoromethyl compound (**40**) showed an excellent inhibitory potency characterized by *K_i_* = 36.3 ± 3.9 pM, making it the best inhibitor within the series of monosubstituted derivatives of Ebselen. The other *para* substitutions were less favored, as manifested by 2–3 orders of magnitude higher *K_i_* values, varying from 1.78 ± 0.18 nM for the structurally extended *n*-butoxy analog **42** to 44.0 ± 4.2 nM for its shorter methoxy homolog **41**.

Studying the kinetics of Ebselen analogs that were disubstituted at the phenyl ring provided the most interesting data (Table 1, right column, **43**–**54**). With antiureolytic activity taken into account, these analogs could be divided into two groups. The first group included most of the disubstituted derivatives, which followed the characteristics of monosubstituted 2-aryl-1,2-benzisoselenazol-3(2*H*)-ones. This meant a very good inhibitory potency expressed by low nanomolar *K_i_* values (1.63–7.30 nM) comparable to that of Ebselen, with the only exception of compound **48** (*K_i_* = 75.2 ± 6.2 nM). The other group constituted the derivatives that were 2,4- and 2,5-dihalogenated at the phenyl ring with chloro and/or fluoro substituents. Incorporation of these structural motives yielded exceptionally active compounds, to the best of our knowledge, the most potent inhibitors of urease reported so far. *K_i_* values for these compounds were in a low picomolar range (5.26–16.7 pM). The highest potency was achieved for 4-chloro-2-fluoro- (**45**, *K_i_* = 5.26 ± 0.41 pM) and 2-chloro-4-fluoro-substituted compounds (**47**, *K_i_* = 5.80 ± 0.40 pM).

**Figure 1:**
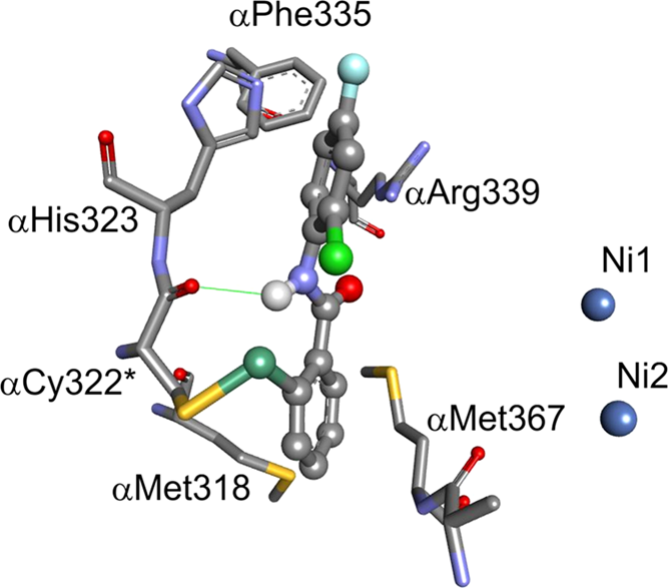
Modeled mode of binding of compound **47** to the urease of *S. pasteurii*. Residues of the active site are shown as sticks, whereas the bound inhibitor is colored in the ball-and-stick representation according to the atom type (gray, carbon; blue, nitrogen; white, hydrogen; red, oxygen; orange, sulfur; light green, chlorine; light blue, fluorine; dark green, selenium; dark blue, nickel). The hydrogen bond is shown as a thin green solid line.

To clarify the significance of F/Cl substitution at the molecular level, the mode of binding of compound **47** to the urease of *S. pasteurii* was modeled, preliminarily assuming the opening of the selenazolone ring and the formation of the covalent Cys-S–Se-Ebselen bond. The overall positioning of the 2-chloro-4-fluoro derivative **47** at the active site of urease (Figure 1) corresponds to that modeled for the reference compound Ebselen.^25^ Accordingly, the NH group of the ligand forms a hydrogen bond with the carbonyl of αCys322* (i.e., the substituted cysteine residue), while the Se-substituted phenyl ring is located in the hydrophobic cleft formed by αMet318 and αMet367. The halogenated aromatic ring of **47** is conveniently sandwiched between the imidazole of αHis323 (edge-to-face) and the guanidinium group of αArg339. Substitution of the aromatic ring with two halogen atoms changes the electron distribution and significantly enhances the π–π interaction with the heteroaromatic side chain of αHis323, as well as the cation−π interactions with αArg339.^46^ These interactions, which are related to the substitution with Cl/F, significantly reflect the free binding energy of an inhibitor and the enzyme and are apparently responsible for an increased inhibitory activity of compounds **44**, **45**, **47**, **49**, and **51** (see also the Supporting Information, Figure S29).

**Figure 2:**
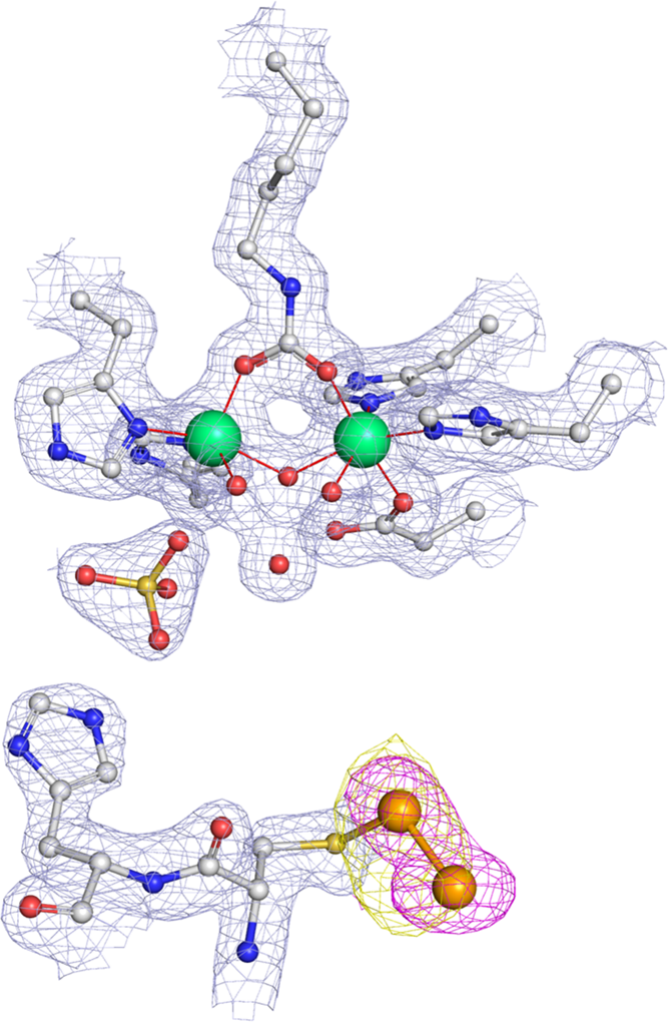
Active site region of the X-ray crystal structure of *S. pasteurii* urease bound to two Se atoms after crystallization in the presence of **47** (PDB id 7ZCY). The atomic model for the protein and the nickel ions is shown superimposed on the final 2*F*_o_ – *F*_c_ electron density Fourier map contoured at 1σ and colored gray, while the two Se atoms are superimposed on the unbiased *F*_o_ – *F*_c_ omit map contoured at the 3σ level and colored in magenta and on the anomalous difference electron density Fourier map contoured at 4σ level and colored in yellow. The carbon, nitrogen, oxygen, sulfur, nickel, and selenium atoms are gray, blue, red, yellow, green, and orange, respectively.

Nonetheless, the structure of the modeled inhibitor–protein complex described above could be considered only as an initial covalent adduct. The complex mode of binding was evidenced in the X-ray crystal structure of SPU cocrystallized in the presence of **47** (deposited in the Protein Data Bank with the accession code 7ZCY; see Table S1 for data collection and final refinement statistics). It shows the typical (αβγ)_3_ quaternary assembly of bacterial ureases and a large conservation of overall folding of the backbone with respect to the native SPU (PDB code 4CEU),^47^ as revealed by the small Cα root-mean-square deviation (RMSD) values calculated for chains α (0.178 Å), β (0.079 Å), and γ (0.077 Å). The Ni-containing active site region is completely conserved with respect to that of the native *S. pasteurii* urease (Tables S1 and S2 and Figure 2), with the dinuclear Ni(1)–Ni(2) cluster (where the two Ni ions are 3.7 Å far apart) being bridged by the Oθ1 and Oθ2 atoms of a carbamoylated αLys220* residue and a hydroxide ion W(B). Ni(1) is also coordinated to αHis249 Nδ and αHis275 Nε, while Ni(2) is bound to αHis137 Nδ, αHis139 Nε, and αAsp363 Oδ1. The active site hydration environment involves three well-ordered water molecules that form, together with the bridging W(B), a pseudo-tetrahedral arrangement of closed-spaced solvent molecules: W(1) and W(2), which complete a distorted square-pyramidal and a distorted octahedral coordination for Ni(1) and Ni(2), respectively, and W(3), located in a distal position and at H-bonding distance from W(B), W(1), and W(2).

**Figure 3:**
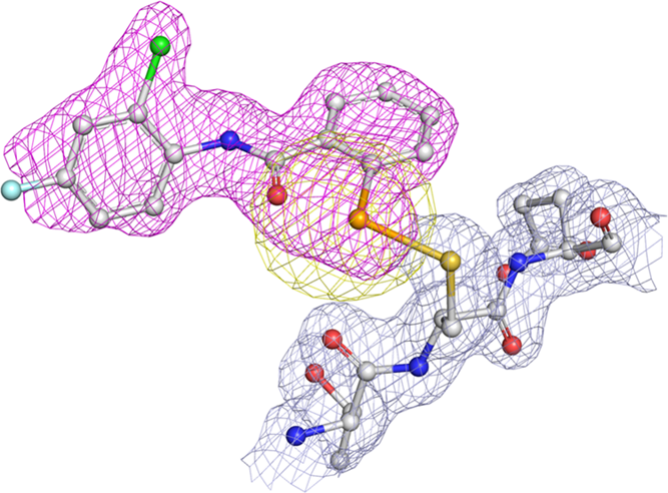
Region proximal to the αCys555* residue of the X-ray crystal structure of *S. pasteurii* urease bound to **47** (PDB id 7ZCY). The atomic model for the protein is shown superimposed on the final 2*F*_o_ – *F*_c_ electron density Fourier map contoured at 1σ and colored gray, while **47** is superimposed on the unbiased *F*_o_ – *F*_c_ omit Fourier map contoured at 3σ and colored in magenta and on the anomalous difference electron density Fourier map contoured at 4σ and colored yellow. The carbon, nitrogen, oxygen, sulfur, and selenium atoms are gray, blue, red, yellow, and orange, respectively.

The unbiased omit electron density Fourier map calculated before the addition of the ligands in the refined model revealed two positive and unmodeled regions in proximity of the two solvent-exposed cysteine residues of SPU, namely, αCys322 and αCys555, thus suggesting covalent adducts formed on the Sγ atoms of those residues (Figures 2 and 3).

The unmodeled electron density peak located next to αCys322* revealed a peculiar oblong shape, suggesting the presence of two spherical ligands possibly forming a dinuclear Se cluster bound to the Sγ atom of αCys322* (Figure 2). Similarly, the anomalous difference electron density Fourier map showed an oblong shape overlapping to the omit map, confirming the attribution of two Se atoms, which were therefore both successfully modeled and refined with a 70% occupancy: Se(1) was refined bound to αCys322* Sγ at *ca.* 2.2 Å, while Se(2) was refined bound to Se(1) at *ca.* 2.3 Å. Table S3 reports a complete list of distances and angles around the Se atoms. A comparison with the same structural parameters obtained using quantum mechanics density functional theory (DFT) calculations on the neutral or the anionic Me–S–Se–Se moiety, also shown in Table S3, supports the crystallographic interpretation of the electron density maps and favors the presence of the anionic form, which features S–Se and Se–Se distances most consistent with those observed experimentally. The differences in angles and dihedrals might be more influenced by the local protein environment and are thus considered less indicative. However, the neutral Cys-S–Se–Se–H form cannot be excluded.

The electron density proximal to αCys555* revealed a different kind of binding (Figure 3). Here, the *F*_o_ – *F*_c_ omit Fourier map region closest to the αCys555* Sγ atom completely overlapped with a complete ligand adduct characterized by a strong anomalous signal of spherical shape and compatible with the presence of a single Se atom bridging αCys555* and the adduct. The electron density was successfully interpreted by modeling **47** with full occupancy and bound to the αCys555* Sγ atom through its Se atom (at *ca.* 2.4 Å).

The mobile flap region of the determined X-ray crystal structure of SPU bound to two Se atoms was found in the open state, with a mainly conserved protein backbone conformation with respect to that of the native enzyme (Figure S30). This overall state is similar to that observed in the case of the crystal structure SPU bound to 1,4-benzoquinone,^48^ catechol,^36^ and its derivatives.^40^

The expected formation of the S–Se adduct at solvent-exposed thiols of αCys322 and αCys555^1,9,25^ was followed by an unexpected secondary reaction that resulted in the formation of an S–Se–Se adduct at αCys322 as a urease inhibitor complex structure. This reactivity involves exclusively αCys322 and not αCys555, suggesting a role for the adjacent αHis323 residue, and implies that the initial adduct is hydrolyzed within the active site with the assistance of the acid–base proton transfer mediated by the αCys322−αHis323 dyad. Such a catalytic mechanism was recently proposed for the inactivation of M^pro^, the SARS-CoV-2 main protease, with Ebselen.^49^ The presence of two Se atoms in the structure of SPU inhibited by the Ebselen derivative indicates that for urease αCys322 two consecutive reaction cycles occur. The two consecutive reaction cycles lead to the overall diselenation of the thiolate group of αCys322. The favorable binding of reactive halogenated inhibitors at the active site and the particularly suitable nearby presence of αHis323 apparently facilitate the repetition of the two-step process. Such an unusual chemical behavior may also clarify the remarkable inhibitory potency of compounds **44, 45, 47, 49**, and **51** and their specificity for urease.

Estimation of the extent of urease inhibition in living cells of *P. mirabilis* PCM 543 was preliminarily carried out under nongrowth conditions in phosphate-buffered saline (PBS). All compounds were proven to be diffusible and efficient inhibitors, with IC_50_ values moderately differentiated in the range between 4.19 ± 0.45 (**35**) and 54.0 ± 2.7 nM (**50**) (Table 2). Some structures exerted a several-fold higher effect than the reference Ebselen (**1**, IC_50_ = 29.2 ± 3.1 nM). These included Ebselen derivatives that were monofluoro-derived at the *ortho* (**31**) and *meta* (**36**) positions of the phenyl ring and the analogous pair of methoxy derivatives **35** and **37**. Disubstituted compounds were found somewhat less potent, with IC_50_ still at an impressive level of 12–25 nM. Inhibition of urease activity exhibited by di(fluorinated/chlorinated) compounds in an isolated enzyme assay was also observed in cells.

**Table 2:** Activity of Selected Urease Inhibitors against Urea Decomposition by *P. mirabilis* PCM 543 (the Most Significant Activity Indicated in Bold)

entry	IC_50_ [nM] (PBS buffer)	IC_90_ [nM] (urine model)	entry	IC_50_ [nM] (PBS buffer)	IC_90_ [nM] (urine model)
**1**	29.2 ± 3.1	112 ± 12			
**30**	25.0 ± 2.0	121 ± 13	**43**	25.1 ± 2.0	148 ± 10
**31**	**7.48** ± **0.67**	**34.7** ± **2.7**	**44**	17.0 ± 1.8	130 ± 16
**32**	13.6 ± 1.5	87.7 ± 8.3	**45**	24.6 ± 2.8	361 ± 21
**33**	26.9 ± 2.8	543 ± 26	**46**	20.0 ± 1.7	176 ± 12
**34**	16.8 ± 1.4	148 ± 6	**47**	22.7 ± 2.5	126 ± 8
**35**	**4.19** ± **0.45**	**18.8** ± **2.4**	**49**	11.8 ± 1.0	151 ± 20
**36**	**8.86** ± **0.81**	223 ± 17	**50**	54.0 ± 2.7	243 ± 17
**37**	11.5 ± 1.3	53.8 ± 3.7	**51**	21.2 ± 1.5	254 ± 14
**39**	12.1 ± 1.4	145 ± 17	**52**	11.8 ± 1.6	96.6 ± 8.1
**40**	16.0 ± 1.3	100 ± 14	**53**	21.9 ± 1.7	82.2 ± 11.3
**41**	15.9 ± 1.1	180 ± 10	**54**	23.8 ± 2.0	124 ± 14
**42**	32.2 ± 3.8	224 ± 15			

The potency of the inhibitors was further characterized under more physiological conditions. *P. mirabilis* PCM 543 cells were incubated in artificial urine medium containing urea as the sole nitrogen source and glucose as the carbon and energy sources. The IC_90_ parameter was estimated to reveal the concentrations required for the almost complete inhibition of ureolysis (Table 2). The IC_90_ values determined in artificial urine ranged from 18.8 ± 2.4 nM for the most active inhibitor **35** (*o*-OMe) to 543 ± 26 nM for the least effective derivative of *o*-Br Ebselen **33**. Six compounds of the 24 tested showed this parameter below 100 nM. The activity pattern was similar to that observed in the PBS buffer with the difference that *ortho* substitutions in the Ebselen phenyl ring were more efficient modifications compared to *meta* isomers (IC_90_ = 34.7 ± 2.7 nM for compound **31** and 223 ± 17 nM for **36**, for example).

Changes in pH were followed in the time course of incubation of *P. mirabilis* PCM 543 in urine medium in the presence of urease inhibitors at a specific concentration of IC_90_. In the case of each compound studied, the increase did not exceed 0.5 units compared to the untreated control samples in which the pH increased from 6.5 to 8.7 in 3 h. This observation confirmed that the inhibitors could stabilize artificial urine despite the content of live *Proteus* cells. To exclude the possibility that the observed effects could occur due to cell decay rather than urease inhibition, a standard MTT (3-(4,5-dimethylthiazol-2-yl)-2,5-diphenyltetrazolium bromide) viability assay was performed with cells exposed to Ebselen-based inhibitors. The results of the MTT assay did not indicate significant differences in the content of viable cells in samples exposed to compounds at IC_90_ concentrations and in control samples; also, the conditions applied for the ureolysis assays did not have a negative effect on *Proteus* cells.

## Conclusions

The previously reported discovery of low nanomolar inhibitory activity of Ebselen against bacterial urease^25^ was a starting point for the structural optimization presented in this work. SAR analysis of the Ebselen scaffold mono/disubstituted in the phenyl ring (25 derivatives) revealed urease inhibitors of exceptional activity, exceeding the reference molecule by 3 orders of magnitude. The most potent compounds showed low picomolar *K_i_* values and comprised a double fluorine/chlorine substitution at positions 2,4 or 2,5 of the phenyl ring. Halogenation has a multidimensional impact on drug potency, lipophilicity, permeability, and metabolic stability and is reflected in the broad applications of halogenated compounds in medicinal chemistry.^50,51^ The typical influence on ligand binding, related to high electronegativity and electron-withdrawing properties, involves the modulation of hydrogen bonding and electrostatic interactions but rather marginal steric effects. In fact, the modeling studies performed (assuming the preliminary formation of a covalent S–Se complex) showed increased π–π and π–cation interactions of the electron-deficient aromatic fragment of the most active derivatives with the enzyme residues, compared to the unsubstituted phenyl ring of the reference compound **1**.

The thiolate functionalities of the two solvent-available residues of urease (αCys322 and αCys555) reacted by opening the isoselenazolone ring of the inhibitors. Structural studies revealed one location (αCys555*) where the adduct remained intact as previously envisioned.^25^

However, this initial complex was hydrolyzed in the case of αCys322*, and this different reactivity was associated with the presence of a neighboring histidine residue (αHis323) with the consequent ability to perform acid–base catalytic activity to obtain the mono-selenated urease-αCys322*-S–Se moiety. Recurrence of this reaction sequence within the active site led to the observed diselenated moiety αCys322*-S–Se–Se, which underscored a novel mechanism of action. This mode of action must be specific for urease and for other cases in which solvent-exposed cysteines, surrounded by protein environments that favor the association with Ebselen to produce the initial adduct, are also located in the near-proximity of a histidine residue able to carry out the necessary acid–base catalysis.

For a range of benzisoselenazolones, the high antiureolytic activity was further confirmed in living cells of *P. mirabilis* and an artificial urine model. Although in these cases the SAR data was somewhat flattened, the values of IC_50_ and IC_90_ remained at an impressive nanomolar level. The permeation of antimicrobials is a major issue in Gram-negative species due to the unique structural and functional complexity of the cell envelope. To reach the cytoplasmic target, the chemical needs to cross the asymmetric bilayer of the outer membrane, pass through the phospholipid inner membrane, and evade the action of the efflux system upregulated in an immediate cell response to harmful agents. The components of the cell protection system differ in the mode of retarding compounds with respect to the physicochemical characteristics of the molecule, making permeation highly selective.^52^ This may be the reason for the different pattern of inhibition of urease observed in whole cells of *P. mirabilis* compared to the susceptibility of the pure enzyme; nevertheless, each studied inhibitor permeated *P. mirabilis* and exerted a highly satisfactory effect on the enzyme located in the cytoplasm.

## Experimental Section

### Chemistry and General Methods

All reagents used were purchased from commercial suppliers, Merck Poland—Sigma-Aldrich and Avantor Performance Materials Poland, and were mostly used without further purification. Anhydrous acetonitrile and methylene chloride were obtained by distillation of a commercially available solvent of analytical grade over P_2_O_5_. The distillated triethylamine was stored in NaOH pellets. Reactions were monitored by thin-layer chromatography (TLC) carried out on 0.25 mm silica gel plates with a fluorescent label (silica gel 60F254), and the components were visualized using the following methods: ultraviolet (UV) light absorption and/or incubation with iodine. Purification of benzisoselenazol-3(2*H*)-ones **34** and **42** by column chromatography was carried out on Merck Si60 silica gel (70–230 mesh), eluted with the indicated solvents. The melting points were determined on an Electrothermal IA 91100 digital melting point apparatus using the standard open capillary method. The ^1^H, ^13^C, and ^77^Se NMR spectra were recorded in CDCl_3_ or DMSO-*d*_6_ on a Bruker Avance DRX 300, Bruker Avance II 600, or Jeol ECZ 400S spectrometer at frequencies 300.1, 600.6, or 399.8 MHz (^1^H), 75.4, 151.0, or 100.5 MHz (^13^C), and 57.2, 114.5, or 76.2 MHz (^77^Se), respectively, at 295 K. Chemical shifts were reported in parts per million (ppm, δ) downfield from tetramethylsilane. Residual solvent central signals were recorded as follows: CDCl_3_, δ_H_ = 7.263, δ_C_ = 77.00; DMSO-*d*_6_, δ_H_ = 2.50, δ_C_ = 39.43. Proton coupling patterns were described as singlet (s), doublet (d), triplet (t), quartet (q), and multiplet (m). High-resolution mass spectra (HRMS) were recorded using an electron spray ionization (ESI) technique on a Waters LCT Premier XE spectrometer. Analytical reverse-phase high-performance liquid chromatography was performed using the UFLC Shimadzu system and Kromasil 100-5-C18, 4.6 mm × 150 mm or Reprosil Saphir 100 C18 column, 4.6 mm × 150 mm (10 → 90% B, 45 min, conditions I; 20 → 90% B, 35 min, conditions II; 20 → 90% B, 30 min, conditions III), flow 0.9 mL/min. Chromatograms were recorded at wavelengths of 222 and 254 nm. Solvent A: 0.1% TFA in water, solvent B: 0.1% TFA in acetonitrile.

2,2′-Diselenobisbenzoic acid (**3**) was prepared from anthranilic acid (**2**), using dilithium diselenide, while 2-(chloroseleno)benzoyl chloride (**4**) was prepared from **3**, as previously described.^12,44^

All synthesized compounds gave satisfactory NMR spectra and HRMS analysis. The final 2-aryl-substituted 1,2-benzisoselenazol-3(2*H*)-ones **30**–**54** were >95% pure, as confirmed by analytical reverse-phase high-performance liquid chromatography (for the high-performance liquid chromatography (HPLC) traces, see the Supporting Information). These compounds were fully characterized in our previous articles^12,44,45,53^ and/or the works presented by other authors.^54−57^ The new compound **47** was fully characterized here.

#### General Procedure of the Synthesis of 2-Aryl-Substituted 1,2-Benzisoselenazol-3(2*H*)-ones (**30**–**54**)^12,44^

2-(Chloroseleno)benzoyl chloride (**4**, 1.27 g, 5.0 mmol) was dissolved in anhydrous acetonitrile or dichloromethane (25 mL) and slowly added dropwise (during *ca.* 1 h) to the stirred solution of aniline (**5**–**29**, 5.0 mmol) and dry triethylamine (1.8 mL, 1.02 g, 12.5 mmol) in anhydrous acetonitrile or dichloromethane (50 mL). The reaction mixture was stirred for 1–48 h, as long as the presence of chloride **4** was not indicated on TLC. Subsequently, the reaction mixture was concentrated under reduced pressure and water (100 mL) was added dropwise during stirring to dissolve triethylamine hydrochloride formed and precipitate the product. The crude benzisoselenazolone **30**–**54** was washed with water and 1 M HCl (to remove triethylamine and unreacted aniline) and with water, dried (on the air and at vesicatory under P_2_O_5_ (20 mmHg)), and if necessary recrystallized from the indicated solvent, except products **34** and **42**. The crude dark purplish-blue materials **34** and **42** were purified by silica gel column chromatography eluted with CH_2_Cl_2_ or AcOEt, respectively, before crystallization.

#### 2-(2-Methylphenyl)-1,2-benzisoselenazol-3(2*H*)-one (**30**)^54,57^

Pale yellow prisms, yield 62%, mp 189–191 °C (MeCN:H_2_O, 2:1, v/v). ^1^H NMR (399.8 MHz, DMSO-*d*_6_) δ 2.11 (s, 3H), 7.37–7.27 (m, 4H), 7.48 (ddd, *J* = 7.8, 7.2, 1.0 Hz, 1H), 7.68 (ddd, *J* = 8.1, 7.2, 1.2 Hz, 1H), 7.90 (dd, *J* = 7.8, 1.2 Hz, 1H), 8.10 (d, *J* = 8.1 Hz, 1H). ^13^C NMR (100.5 MHz, DMSO-*d*_6_) δ 17.79, 125.92, 126.08, 126.67, 127.27, 127.87, 128.13, 128.73, 130.73, 131.93, 136.55, 137.37, 140.12, 164.04. ^77^Se NMR (76.2 MHz, DMSO-*d*_6_) δ 917.69. HRMS (TOF MS ESI) *m*/*z* calcd for C_14_H_11_NOSe + H^+^ 290.0084; found 290.0094.

#### 2-(2-Fluorophenyl)-1,2-benzisoselenazol-3(2*H*)-one (**31**)^55,57^

Yellow solid, yield 65%, mp 163–164 °C (AcOEt). ^1^H NMR (399.8 MHz, DMSO-*d*_6_) δ 7.30 (ddd, *J* = 7.9, 7.2, 1.5 Hz, 1H), 7.38 (ddd, *J* = 10.2, 8.3, 1.5 Hz, 1H), 7.45 (dddd, *J* = 8.3, 7.3, 5.3, 1.6 Hz, 1H), 7.49 (ddd, *J* = 7.7, 7.2, 1.4 Hz, 1H), 7.51 (ddd, *J* = 8.0, 7.8, 1.4 Hz, 1H), 7.70 (ddd, *J* = 8.1, 7.3, 1.4 Hz, 1H), 7.91 (dd, *J* = 7.7, 1.4 Hz, 1H), 8.10 (d, *J* = 8.0 Hz, 1H). ^13^C NMR (100.5 MHz, DMSO-*d*_6_) δ 116.50 (d, *J* = 19.8 Hz), 124.89 (d, *J* = 3.2 Hz), 126.01, 126.19, 126.28 (d, *J* = 17.3 Hz), 126.83, 127.93, 129.47 (d, *J* = 7.8 Hz), 130.18, 132.27, 140.28, 157.61 (d, *J* = 250.0 Hz), 165.57. ^77^Se NMR (76.2 MHz, DMSO-*d*_6_) δ 940.51 (d, *J* = 18.2 Hz). HRMS (TOF MS ESI) *m*/*z* calcd for C_13_H_8_FNOSe + H^+^ 293.9833; found 293.9838.

#### 2-(2-Chlorophenyl)-1,2-benzisoselenazol-3(2*H*)-one (**32**)^55^

Pale yellow crystals, yield 75%, mp 200–203 °C. ^1^H NMR (399.8 MHz, DMSO-*d*_6_) δ 7.43–7.47 (m, 2H), 7.49 (ddd, *J* = 7.7, 7.3, 0.9 Hz, 1H), 7.49–7.53 (m, 1H), 7.60–7.65 (m, 1H), 7.70 (ddd, *J* = 8.0, 7.3, 1.3 Hz, 1H), 7.90 (dd, *J* = 7.7, 0.9 Hz, 1H), 8.10 (d, *J* = 8.0 Hz, 1H). ^13^C NMR (100.5 MHz, DMSO-*d*_6_) δ 125.99, 126.12, 126.80, 127.94, 128.05, 129.75, 130.12, 131.12, 132.21, 132.53, 136.15, 140.35, 165.59. ^77^Se NMR (76.2 MHz, DMSO-*d*_6_) δ 936.61. HRMS (TOF MS ESI) *m*/*z* calcd for C_13_H_8_ClNOSe + H^+^ 309.9538; found 309.9531.

#### 2-(2-Bromophenyl)-1,2-benzisoselenazol-3(2*H*)-one (**33**)^55^

Pale yellow prisms, yield 65%, mp 214–216 °C. ^1^H NMR (600.6 MHz, DMSO-*d*_6_) δ 7.37 (ddd, *J* = 8.1, 6.3, 2.8 Hz, 1H), 7.47–7.51 (m, 3H), 7.69 (ddd, *J* = 8.1, 7.2, 1.4 Hz, 1H), 7.77 (dd, *J* = 8.1, 1.0 Hz, 1H), 7.91 (ddd, *J* = 7.7, 1.4, 0.6 Hz, 1H), 8.10 (d, *J* = 8.0 Hz, 1H). ^13^C NMR (100.5 MHz, DMSO-*d*_6_) δ 123.23, 125.96, 126.08, 126.88, 127.95, 128.64, 130.02, 131.22, 132.18, 133.22, 137.75, 140.31, 165.50. ^77^Se NMR (76.2 MHz, DMSO-*d*_6_) δ 935.53. HRMS (TOF MS ESI) *m*/*z* calcd for C_13_H_8_BrNOSe + H^+^ 353.9033; found 353.9026.

#### 2-(2-Hydroxyphenyl)-1,2-benzisoselenazol-3(2*H*)-one (**34**)^12,53,56^

Orange flakes, yield 40%, mp 195.5–197.5 °C (MeCN:H_2_O, 6:1, v/v). ^1^H NMR (399.8 MHz, CDCl_3_) δ 6.99 (ddd, *J* = 8.0, 7.2, 1.5 Hz, 1H), 7.13 (dd, *J* = 8.6, 1.5 Hz, 1H), 7.24–7.30 (m, 2H), 7.47–7.54 (m, 1H), 7.65–7.71 (m, 2H), 8.13 (d, *J* = 8.1 Hz, 1H), 8.55 (s, 1H). ^13^C NMR (100.5 MHz, DMSO-*d*_6_) δ 116.94, 119.22, 125.73, 125.81, 125.97, 127.52, 127.76, 128.76, 129.32, 131.82, 140.50, 153.25, 165.76. ^77^Se NMR (114.5 MHz, DMSO-*d*_6_) δ 930.02. HRMS (TOF MS ESI) *m*/*z* calcd for C_13_H_9_NO_2_Se + Na^+^ 313.9696; found 313.9688.

#### 2-(2-Methoxyphenyl)-1,2-benzisoselenazol-3(2*H*)-one (**35**)^12,53−55^

Yellow prisms, yield 71%, mp 189.0–191.5 °C (CHCl_3_). ^1^H NMR (300.1 MHz, CDCl_3_) δ 3.85 (s, 3H), 6.98–7.07 (m, 2H), 7.36 (ddd, *J* = 8.2, 7.6, 1.7 Hz, 1H), 7.44 (ddd, *J* = 8.0, 6.4, 1.7 Hz, 1H), 7.48 (dd, *J* = 8.0, 1.7 Hz, 1H), 7.58–7.70 (m, 2H), 8.13 (d, *J* = 7.8 Hz, 1H). ^13^C NMR (75.4 MHz, CDCl_3_) δ 55.80, 112.22, 120.74, 123.86, 126.01, 126.44, 126.74, 129.21, 129.66, 129.89, 132.17, 139.31, 155.39, 166.70. ^77^Se NMR (114.5 MHz, DMSO-*d*_6_) δ 946.31. HRMS (TOF MS ESI) *m*/*z* calcd for C_14_H_11_NO_2_Se + H^+^ 306.0033; found 306.0041.

#### 2-(3-Fluorophenyl)-1,2-benzisoselenazol-3(2*H*)-one (**36**)^12,56^

Pale yellow needles, yield 65%, mp 190.5–191.0 °C (H_2_O:MeCN, 4:3, v/v). ^1^H NMR (600.6 MHz, DMSO-*d*_6_) δ 7.11 (dddd, *J* = 8.4, 8.4, 2.6, 1.0 Hz, 1H), 7.44 (ddd, *J* = 8.1, 7.4, 1.0 Hz, 1H), 7.47–7.53 (m, 2H), 7.66–7.75 (m, 2H), 7.93 (ddd, *J* = 7.7, 1.5, 0.6 Hz, 1H), 8.10 (d, *J* = 8.1 Hz, 1H). ^13^C NMR (151.0 MHz, DMSO-*d*_6_) δ 111.18 (d, *J* = 25.1 Hz), 112.30 (d, *J* = 21.2 Hz), 120.06 (d, *J* = 2.7 Hz), 125.86, 126.36, 128.04, 128.49, 130.83 (d, *J* = 9.4 Hz), 132.54, 138.79, 141.50 (d, *J* = 10.7 Hz), 162.11 (d, *J* = 243.6 Hz), 165.26. ^77^Se NMR (114.5 MHz, DMSO-*d*_6_) δ 920.36. HRMS (TOF MS ESI) *m*/*z* calcd for C_13_H_8_FNOSe + H^+^ 293.9833; found 293.9839.

#### 2-(3-Methoxyphenyl)-1,2-benzisoselenazol-3(2*H*)-one (**37**)^44,53,56,57^

Yellow crystals, yield 65%, mp 166–168 °C (MeCN:H_2_O, 1:1, v/v). ^1^H NMR (600.6 MHz, DMSO-*d*_6_) δ 3.79 (s, 3H), 6.86 (ddd, *J* = 8.3, 2.5, 0.7 Hz, 1H), 7.16 (ddd, *J* = 7.9, 2.0, 0.7 Hz, 1H), 7.32 (dd, *J* = 2.5, 2.0 Hz, 1H), 7.36 (dd, *J* = 8.3, 7.9 Hz, 1H), 7.48 (ddd, *J* = 7.7, 7.2, 0.9 Hz, 1H), 7.68 (ddd, *J* = 8.0, 7.2, 1.4 Hz, 1H), 7.91 (dd, *J* = 7.7, 0.9 Hz, 1H), 8.08 (d, *J* = 8.0 Hz, 1H). ^13^C NMR (151.0 MHz, DMSO-*d*_6_) δ 55.17, 110.29, 111.27, 116.58, 125.71, 126.18, 127.86, 128.53, 129.91, 132.20, 138.79, 140.78, 159.58, 164.93. ^77^Se NMR (76.2 MHz, DMSO-*d*_6_) δ 916.40. HRMS (TOF MS ESI) *m*/*z* calcd for C_14_H_11_NO_2_Se + H^+^ 306.0033; found 306.0030.

#### 2-(4-Methylphenyl)-1,2-benzisoselenazol-3(2*H*)-one (**38**)^12,44,53,54,57^

Pale powder, yield 72%, mp 163–164 °C (H_2_O). ^1^H NMR (300.1 MHz, DMSO-*d*_6_) δ 2.32 (s, 3H), 7.24 (d, *J =* 8.1 Hz, 2H), 7.47–7.53 (m, 3H), 7.67 (ddd, *J =* 8.0, 7.5, 1.3 Hz, 1H), 7.90 (d, *J =* 7.0 Hz, 1H), 8.09 (d, *J =* 8.0 Hz, 1H). ^13^C NMR (75.4 MHz, DMSO-*d*_6_) δ 20.5, 124.5, 125.7, 126.2, 127.8, 128.4, 129.5, 132.0, 135.1, 137.0, 138.8, 164.8. ^77^Se NMR (114.5 MHz, DMSO-*d*_6_) δ 925.64. HRMS (TOF MS ESI) *m*/*z* calcd for C_14_H_11_NOSe + H^+^ 290.0085; found 290.0084.

#### 2-(4-Fluorophenyl)-1,2-benzisoselenazol-3(2*H*)-one (**39**)^12,56,57^

Pale yellow plates, yield 70%, mp 177–178 °C (MeCN:H_2_O, 1:1, v/v). ^1^H NMR (600.6 MHz, DMSO-*d*_6_) δ 7.30 (dd, *J* = 8.8, 8.8 Hz, 2H), 7.49 (ddd, *J* = 8.0, 7.2, 1.0 Hz, 1H), 7.66 (dd, *J* = 9.0, 4.9 Hz, 2H), 7.69 (ddd, *J* = 8.2, 7.2, 1.4 Hz, 1H), 7.92 (ddd, *J* = 7.7, 1.4, 0.6 Hz, 1H), 8.10 (d, *J* = 8.1 Hz, 1H). ^13^C NMR (151.0 MHz, DMSO-*d*_6_) δ 115.91 (d, *J* = 22.5 Hz), 125.86, 126.29, 126.93 (d, *J* = 8.3 Hz), 127.98, 128.21, 132.29, 135.89 (d, *J* = 2.8 Hz), 138.93, 159.72 (d, *J* = 243.5 Hz), 165.12. ^77^Se NMR (114.5 MHz, DMSO-*d*_6_) δ 919.76. HRMS (TOF MS ESI) *m*/*z* calcd for C_13_H_8_FNOSe + H^+^ 293.9833; found 293.9822.

#### 2-(4-Trifluoromethylphenyl)-1,2-benzisoselenazol-3(2*H*)-one (**40**)^57^

Bright yellow flakes, yield 54%, mp 241–242 °C (AcOEt). ^1^H NMR (600.6 MHz, DMSO-*d*_6_) δ 7.50 (ddd, *J* = 7.7, 7.5, 0.9 Hz, 1H), 7.71 (ddd, *J* = 8.0, 7.5, 1.4 Hz, 1H), 7.80 (d, *J* = 8.5 Hz, 2H), 7.94 (dd, *J* = 7.7, 1.4 Hz, 1H), 7.94 (d, *J* = 8.5 Hz, 2H), 8.10 (dd, *J* = 8.0, 0.9 Hz, 1H). ^13^C NMR (100.5 MHz, DMSO-*d*_6_) δ 124.08 (q, *J* = 271.8 Hz), 124.15, 125.24 (q, *J* = 32.3 Hz), 125.84, 126.25 (q, *J* = 3.7 Hz), 126.37, 128.07, 128.37, 132.64, 138.63, 143.59, 165.37. ^77^Se NMR (76.2 MHz, DMSO-*d*_6_) δ 919.59. HRMS (TOF MS ESI) *m*/*z* calcd for C_14_H_8_F_3_NOSe + H^+^ 343.9801; found 343.9794.

#### 2-(4-Methoxyphenyl)-1,2-benzisoselenazol-3(2*H*)-one (**41**)^12,44,54,57^

Pale needles, yield 70%, mp 180.5–181.5 °C (H_2_O). ^1^H NMR (300.1 MHz, DMSO-*d*_6_) δ 3.78 (s, 3H), 7.01 (dd, *J =* 9.0, 2.3 Hz, 2H), 7.47 (ddd, *J =* 7.8, 7.1, 1.0 Hz, 1H), 7.50 (dd, *J =* 9.0, 2.3 Hz, 2H), 7.67 (ddd, *J =* 7.9, 7.2, 1.4 Hz, 1H), 7.89 (dd, *J =* 7.8, 0.8 Hz, 1H), 8.08 (d, *J =* 7.9 Hz, 1H). ^13^C NMR (75.4 MHz, DMSO-*d*_6_) δ 55.3, 114.3, 125.7, 126.1, 126.5, 127.8, 128.2, 131.9, 132.1, 138.9, 157.2, 164.9. ^77^Se NMR (114.5 MHz, DMSO-*d*_6_) δ 915.32. HRMS (TOF MS ESI) *m*/*z* calcd for C_14_H_11_NO_2_Se + H^+^ 306.0033; found 306.0039.

#### 2-(4-*n*-Butoxyphenyl)-1,2-benzisoselenazol-3(2*H*)-one (**42**)^12^

Purple powder, yield 75%, mp 138–140 °C. ^1^H NMR (600.6 MHz, DMSO-*d*_6_) δ 0.92 (t, *J* = 7.4 Hz, 3H), 1.43 (qt, *J* = 7.4, 7.4 Hz, 2H), 1.69 (tt, *J* = 7.4, 6.5 Hz, 2H), 3.97 (t, *J* = 6.5 Hz, 2H), 6.98 (d, *J* = 8.8 Hz, 2H), 7.45–7.50 (m, 3H), 7.66 (dd, *J* = 7.7, 7.5 Hz, 1H), 7.89 (d, *J* = 7.7 Hz, 1H), 8.09 (d, *J* = 8.1 Hz, 1H). ^13^C NMR (151.0 MHz, DMSO-*d*_6_) δ 14.17, 19.23, 31.20, 67.89, 115.30, 126.28, 126.64, 126.96, 128.34, 128.83, 132.48, 132.60, 139.47, 157.24, 165.43. ^77^Se NMR (114.5 MHz, DMSO-*d*_6_): δ 914.35. HRMS (TOF MS ESI) *m*/*z* calcd for C_17_H_17_NO_2_Se + H^+^ 348.0504; found 348.0505.

#### 2-(4-Methoxy-2-methylphenyl)-1,2-benzisoselenazol-3(2*H*)-one (**43**)^12,44^

Beige crystals, yield 66%, mp 179–180 °C (MeCN:H_2_O, 1:1, v/v). ^1^H NMR (300.1 MHz, DMSO-*d*_6_) δ 2.07 (s, 3H), 3.78 (s, 3H), 6.84 (dd, *J =* 8.6, 2.9 Hz, 1H), 6.92 (d, *J =* 2.9 Hz, 1H), 7.20 (d, *J =* 8.6 Hz, 1H), 7.47 (dd, *J =* 8.3, 6.9 Hz, 1H), 7.67 (ddd, *J =* 8.0, 6.9, 1.3 Hz, 1H), 7.88 (dd, *J =* 8.3, 1.3 Hz, 1H), 8.08 (d, *J =* 8.0 Hz, 1H). ^13^C NMR (75.4 MHz, DMSO-*d*_6_) δ 18.0, 55.2, 111.9, 115.7, 125.9, 126.0, 127.3, 127.8, 129.7, 129.9, 131.8, 137.9, 140.0, 158.8, 165.2. ^77^Se NMR (114.5 MHz, DMSO-*d*_6_) δ 911.59. HRMS (TOF MS ESI) *m*/*z* calcd for C_15_H_13_NO_2_Se + H^+^ 320.0190; found 320.0210.

#### 2-(2,4-Difluorophenyl)-1,2-benzisoselenazol-3(2*H*)-one (**44**)^45^

Pale yellow crystals, yield 75%, mp 175–176 °C (AcOEt). ^1^H NMR (600.6 MHz, DMSO-*d*_6_) δ 7.20 (dddd, *J* = 8.8, 8.2, 2.9, 1.2 Hz, 1H), 7.45 (ddd, *J* = 10.3, 9.2, 2.9 Hz, 1H), 7.49 (ddd, *J* = 7.7, 7.2, 0.9 Hz, 1H), 7.56 (ddd, *J* = 8.8, 8.8, 6.1 Hz, 1H), 7.70 (ddd, *J* = 8.0, 7.2, 1.4 Hz, 1H), 7.91 (dd, *J* = 7.7, 1.4 Hz, 1H), 8.10 (d, *J* = 8.0 Hz, 1H). ^13^C NMR (100.5 MHz, DMSO-*d*_6_) δ 105.06 (dd, *J* = 25.9, 25.2 Hz), 111.94 (dd, *J* = 22.4, 3.2 Hz), 122.87 (dd, *J* = 13.2, 3.5 Hz), 126.04, 126.22, 126.65, 127.94, 131.47 (d, *J* = 9.3 Hz), 132.34, 140.36, 157.90 (dd, *J* = 252.5, 13.0 Hz), 161.16 (dd, *J* = 247.4, 11.5 Hz), 165.78. ^77^Se NMR (76.2 MHz, DMSO-*d*_6_) δ 942.50 (d, *J* = 15.1 Hz). HRMS (TOF MS ESI) *m*/*z* calcd for C_13_H_7_F_2_NOSe + H^+^ 311.9739; found 311.9739.

#### 2-(4-Chloro-2-fluorophenyl)-1,2-benzisoselenazol-3(2*H*)-one (**45**)^45^

Colorless wool, yield 53%, mp 220–221 °C (AcOEt). ^1^H NMR (600.6 MHz, DMSO-*d*_6_) δ 7.39 (ddd, *J* = 8.5, 2.3, 0.8 Hz, 1H), 7.49 (ddd, *J* = 7.7, 7.2, 0.9 Hz, 1H), 7.55 (dd, *J* = 8.5, 8.3 Hz, 1H), 7.63 (dd, *J* = 10.1, 2.3 Hz, 1H), 7.70 (ddd, *J* = 8.1, 7.2, 1.2 Hz, 1H), 7.91 (dd, *J* = 7.7, 1.2 Hz, 1H), 8.09 (dd, *J* = 8.1, 0.9 Hz, 1H). ^13^C NMR (100.5 MHz, DMSO-*d*_6_) δ 117.12 (d, *J* = 23.7 Hz), 125.09 (d, *J* = 3.3 Hz), 125.59 (d, *J* = 13.1 Hz), 126.02, 126.20, 126.60, 127.93, 131.31, 132.35, 132.61 (d, *J* = 9.9 Hz), 140.34, 157.47 (d, *J* = 253.6 Hz), 165.65. ^77^Se NMR (76.2 MHz, DMSO-*d*_6_) δ 946.74 (d, *J* = 19.8 Hz). HRMS (TOF MS ESI) *m*/*z* calculated for C_13_H_7_ClFNOSe + H^+^ 327.9444; found 327.9446.

#### 2-(2-Chloro-4-methylphenyl)-1,2-benzisoselenazol-3(2*H*)-one (**46**)^12,44^

White powder, yield 95%, mp 208–209 °C (AcOEt). ^1^H NMR (300.1 MHz, CDCl_3_) δ 2.36 (s, 3H), 7.25 (dd, *J =* 8.0, 1.1 Hz, 1H), 7.37 (d, *J =* 8.0 Hz, 1H), 7.45–7.50 (m, 2H), 7.69 (ddd, *J =* 8.0, 7.3, 1.3 Hz, 1H), 7.89 (dd, *J =* 7.7, 0.8 Hz, 1H), 8.09 (d, *J =* 8.0 Hz, 1H). ^13^C NMR (151.0 MHz, DMSO-*d*_6_): δ 20.90, 126.51, 126.59, 127.41, 128.44, 129.11, 130.832, 131.18, 132.64, 132.66, 133.92, 140.41, 140.87, 166.17. ^77^Se NMR (114.5 MHz, DMSO-*d*_6_) δ 932.1574. HRMS (TOF MS ESI) *m/z* calcd for C_14_H_10_ClNOSe+H^+^ 323.9694; found 323.9694.

#### 2-(2-Chloro-4-fluorophenyl)-1,2-benzisoselenazol-3(2*H*)-one (**47**)

Pale needles, yield 51%, mp 182–183 °C (AcOEt). ^1^H NMR (399.8 MHz, DMSO-*d*_6_) δ 7.34 (ddd, *J* = 8.6, 8.5, 2.9 Hz, 1H), 7.48 (ddd, *J* = 8.0, 7.3, 1.0 Hz, 1H), 7.57 (dd, *J* = 8.8, 5.8 Hz, 1H), 7.64 (dd, *J* = 8.6, 2.9 Hz, 1H), 7.70 (ddd, *J* = 8.3, 7.2, 1.5 Hz, 1H), 7.90 (dd, *J* = 7.7, 1.4 Hz, 1H), 8.10 (d, *J* = 8.0 Hz, 1H). ^13^C NMR (100.5 MHz, DMSO-*d*_6_) δ 115.30 (d, *J* = 22.3 Hz), 117.51 (d, *J* = 26.2 Hz), 126.17 (d, *J* = 11.2 Hz), 126.76, 128.05, 132.38, 132.60, 132.69, 132.84 (d, *J* = 3.4 Hz), 133.86 (d, *J* = 11.3 Hz), 140.53, 161.22 (d, *J* = 248.8 Hz), 165.90. ^77^Se NMR (76.2 MHz, DMSO-*d*_6_) δ 938.44. HRMS (TOF MS ESI) *m*/*z* calculated for C_13_H_7_ClFNOSe + H^+^ 327.9444; found 327.9421.

#### 2-(5-Chloro-2-methylphenyl)-1,2-benzisoselenazol-3(2*H*)-one (**48**)^44,45^

White powder, yield 51%, mp 190–191 °C (CHCl_3_). ^1^H NMR (300.1 MHz, DMSO-*d*_6_) δ 2.08 (s, 3H), 7.36–7.42 (m, 3H), 7.49 (dd, *J =* 7.6, 7.3 Hz, 1H), 7.69 (ddd, *J =* 8.0, 7.3, 1.3 Hz, 1H), 7.89 (d, *J =* 7.6, 1H), 8.10 (d, *J =* 8.0 Hz, 1H). ^13^C NMR (100.5 MHz, DMSO-*d*_6_) δ 17.28, 125.99, 126.10, 126.99, 127.89, 127.99, 128.57, 130.25, 132.07, 132.19, 135.77, 138.83, 140.26, 165.23. ^77^Se NMR (76.2 MHz, DMSO-*d*_6_) δ 927.66. HRMS (TOF MS ESI) *m*/*z* calcd for C_14_H_10_ClNOSe + H^+^ 323.9694; found 323.9687.

#### 2-(5-Chloro-2-fluorophenyl)-1,2-benzisoselenazol-3(2*H*)-one (**49**)^45^

Colorless wool, yield 36%, mp 197–198 °C (AcOEt). ^1^H NMR (300.1 MHz, DMSO-*d*_6_) δ 7.37–7.54 (m, 3H), 7.65 (dd, *J* = 6.5, 2.5 Hz, 1H), 7.71 (ddd, *J* = 8.1, 7.2, 1.1 Hz, 1H), 7.91 (d, *J* = 7.7 Hz, 1H), 8.10 (d, *J* = 8.1 Hz, 1H). ^13^C NMR (75.4 MHz, DMSO-*d*_6_) δ 118.1 (d, *J* = 21.9 Hz), 126.0, 126.2, 126.6, 127.8 (d, *J* = 15.1 Hz), 128.0, 128.2 (d, *J* = 3.0 Hz), 129.1 (d, *J* = 8.1 Hz), 129.8, 132.4, 140.4, 156.4 (d, *J* = 249.8 Hz), 165.8. ^77^Se NMR (76.2 MHz, DMSO-*d*_6_) δ 952.86 (d, *J* = 25.5 Hz). HRMS (TOF MS ESI) *m*/*z* calcd for C_13_H_7_ClFNOSe + H^+^ 327.9444; found 327.9446.

#### 2-(2-Chloro-5-methylphenyl)-1,2-benzisoselenazol-3(2*H*)-one (**50**)^44,45^

White powder, yield 63%, mp 183–184 °C (CH_2_Cl_2_). ^1^H NMR (300.1 MHz, CDCl_3_) δ 2.35 (s, 3H), 7.16 (d, *J =* 8.2 Hz, 1H), 7.29 (s, 1H), 7.38 (d, *J =* 8.2 Hz, 1H), 7.47 (dd, *J =* 7.6, 6.8 Hz, 1H), 7.62–7.70 (m, 2H), 8.13 (d, *J =* 7.6 Hz, 1H). ^13^C NMR (75.4 MHz, CDCl_3_) δ 20.5, 124.4, 125.8, 126.1, 129.0, 130.0, 130.4, 130.6, 131.4, 132.3, 135.1, 137.8, 139.5, 166.6. ^77^Se NMR (76.2 MHz, DMSO-*d*_6_) δ 936.44. HRMS (TOF MS ESI) *m*/*z* calcd for C_14_H_10_ClNOSe + H^+^ 323.9694; found 323.9607.

#### 2-(2,5-Dichlorophenyl)-1,2-benzisoselenazol-3(2*H*)-one (**51**)^45^

Pale yellow solid, yield 53%, mp 188–189 °C (AcOEt). ^1^H NMR (399.8 MHz, DMSO-*d*_6_) δ 7.49 (dd, *J* = 7.8, 7.2 Hz, 1H), 7.53 (dd, *J* = 8.6, 2.5 Hz, 1H), 7.66 (d, *J* = 2.5 Hz, 1H), 7.66 (d, *J* = 8.6 Hz, 1H), 7.70 (dd, *J* = 8.1, 7.2 Hz, 1H), 7.90 (d, *J* = 7.8 Hz, 1H), 8.10 (d, *J* = 8.1 Hz, 1H). ^13^C NMR (100.5 MHz, DMSO-*d*_6_) δ 126.05, 126.14, 126.58, 127.98, 129.60, 130.96, 131.40, 131.57, 131.82, 132.36, 137.64, 140.59, 165.79. ^77^Se NMR (76.2 MHz, DMSO-*d*_6_) δ 947.54. HRMS (TOF MS ESI) *m*/*z* calcd for C_13_H_7_Cl_2_NOSe + H^+^ 343.9148; found 343.9140.

#### 2-(2-Methoxy-5-methylphenyl)-1,2-benzisoselenazol-3(2*H*)-one (**52**)^12,44^

Pale brown crystals, yield 63%, mp 172–173 °C (H_2_O). ^1^H NMR (300.1 MHz, DMSO-*d*_6_) δ 2.27 (s, 3H), 3.73 (s, 3H), 7.03 (d, *J =* 6.4 Hz, 1H), 7.16–7.18 (m, 2H), 7.45 (ddd, *J =* 7.9, 7.5, 0.8 Hz, 1H), 7.66 (ddd, *J =* 8.2, 7.0, 1.4 Hz, 1H), 7.86 (dd, *J =* 7.7, 0.9 Hz, 1H), 8.06 (d, *J =* 8.0 Hz, 1H). ^13^C NMR (151.0 MHz, DMSO-*d*_6_) δ 20.33, 56.24, 113.01, 126.31, 126.33, 127.37, 127.86, 128.29, 129.88, 129.96, 130.53, 132.35, 140.89, 153.60, 166.22. ^77^Se NMR (114.5 MHz, DMSO-*d*_6_) δ 932.69. HRMS (TOF MS ESI) *m*/*z* calcd for C_15_H_13_NO_2_Se + H^+^ 320.0190; found 320.0201.

#### 2-(5-Chloro-2-methoxyphenyl)-1,2-benzisoselenazol-3(2*H*)-one (**53**)^12,44,57^

Yellow crystals, yield 79%, mp 205–206 °C (AcCN:H_2_O, 85:15, v/v). ^1^H NMR (300.1 MHz, DMSO-*d*_6_) δ 3.79 (s, 3H), 7.19 (d, *J =* 8.9 Hz, 1H), 7.41–7.49 (m, 3H), 7.67 (ddd, *J =* 8.2, 7.2, 1.4 Hz, 1H), 7.87 (dd, *J =* 7.8, 0.8 Hz, 1H), 8.07 (d, *J =* 8.1 Hz, 1H). ^13^C NMR (151.0 MHz, DMSO-*d*_6_) δ 56.63, 114.62, 124.15, 126.34, 126.42, 127.51, 128.38, 129.04, 129.05, 129.66, 132.62, 141.01, 154.58, 166.48. ^77^Se NMR (114.5 MHz, DMSO-*d*_6_) δ 944.94. HRMS (TOF MS ESI) *m*/*z* calcd for C_14_H_10_ClNO_2_Se + H^+^ 339.9644; found 339.9636.

#### 2-(4-Chloro-3-methylphenyl)-1,2-benzisoselenazol-3(2*H*)-one (**54**)^44,45^

Orange crystals, yield 74%, mp 208–209 °C (H_2_O). ^1^H NMR (600.6 MHz, DMSO-*d*_6_) δ 2.37 (s, 3H), 7.47 (d, *J* = 8.6 Hz, 1H), 7.48 (ddd, *J* = 7.7, 7.2, 0.9 Hz, 1H), 7.50 (dd, *J* = 8.6, 2.4 Hz, 1H), 7.64 (d, *J* = 2.4 Hz, 1H), 7.69 (ddd, *J* = 8.0, 7.2, 1.1 Hz, 1H), 7.90 (dd, *J* = 7.7, 1.1 Hz, 1H), 8.09 (d, *J* = 8.0 Hz, 1H). ^13^C NMR (100.5 MHz, DMSO-*d*_6_) δ 19.62, 123.66, 125.79, 126.23, 126.98, 127.90, 128.25, 129.31, 130.04, 132.29, 136.27, 138.49, 138.79, 165.04. ^77^Se NMR (76.2 MHz, DMSO-*d*_6_) δ 919.71. HRMS (TOF MS ESI) *m*/*z* calcd for C_14_H_10_ClNOSe + H^+^ 323.9694; found 323.9692.

## Supporting Information Available

HPLC traces of the final compounds (**30**–**54**); kinetic data with the methodology used to calculate the inhibition constants and the mechanism of the inhibition; details of the measurement of ureolysis inhibition in whole cells of *P. mirabilis*; the molecular modeling protocol; methodology of crystallization of ligand–protein complexes; and data collection and structural determination (PDF)
Molecular formula strings (CSV)

## Abbreviations Used

MTT: 3-(4,5-dimethylthiazol-2-yl)-2,5-diphenyltetrazolium bromide
PBS: phosphate-buffered saline
SPU: *S. pasteurii* urease
