# Supplementary material for: Optimized Ebselen-Based Inhibitors of Bacterial Ureases with Nontypical Mode of Action
Source: J Med Chem. 2023 Jan 20;66(3):2054–63. doi: 10.1021/acs.jmedchem.2c01799 (PMC9923736; doi:10.1021/acs.jmedchem.2c01799)
Supplement: Supplementary file 1 — jm2c01799_si_001.pdf [file jm2c01799_si_001.pdf]

Supporting Information

Optimized ebselen-based inhibitors of bacterial ureases with nontypical mode of action

Katarzyna Macegoniuk,[a] Wojciech Tabor,[a] Luca Mazzei,[b] Michele Cianci,[c] Mirosław Giurg,[d] Kamila Olech,[d] Małgorzata Burda-Grabowska,[d] Rafał Kaleta,[d] Agnieszka Grabowiecka,[a] Artur Mucha,[a] Stefano Ciurli,[b] Łukasz Berlicki*[a]

[a] Department of Bioorganic Chemistry

Wrocław University of Science and Technology

Wybrzeże Wyspiańskiego 27, 50-370 Wrocław (Poland)

E-mail: lukasz.berlicki@pwr.edu.pl

[b] Laboratory of Bioinorganic Chemistry

Department of Pharmacy and Biotechnology (FaBiT)

University of Bologna

Viale Giuseppe Fanin 40, 40138 Bologna (Italy)

[c] Department of Agricultural, Food and Environmental Sciences

Polytechnic University of Marche

Via Brecce Bianche 10, 60131 Ancona (Italy)

[d] Department of Organic and Medicinal Chemistry

Wrocław University of Science and Technology

Wybrzeże Wyspiańskiego 27, 50-370 Wrocław (Poland)

* Corresponding Author

CONTENTS

S1. HPLC analyses S3

S2. Enzymatic studies S16

S2.1. Inhibition studies. Competitive reversible inhibition S16

S2.2. Inhibition studies. Slow binding inhibition S17

S3. Inhibition of ureolysis in whole cells of *Proteus mirabilis* S19

S3.1.* Proteus mirabilis *PCM543 viability control S20

S4. Molecular modeling S20

S5. Crystallization, data collection and structural determination S22

S5.1. Quantum mechanical calculations S23

S7. References S28

S1. HPLC analyses. Reverse phase high-performance liquid chromatography of compounds **30-54** (characterized in the main body of the manuscript, and elsewhere[S1-S9]) was performed using the UFLC Shimadzu system using gradient elution and analytic Kromasil 100-5-C18 column, 4.6×150 mm (10→90% B, 45 min, conditions **I**) or analytic Reprosil Saphir 100 C18 column, 4.6×150 mm (20→90% B, 35 min, conditions **II**, 30 min, conditions **III**), flow 0.9 mL/min. The chromatograms were recorded at wavelengths 222 and 254 nm.

Solvent A: 0.1% TFA in water, solvent B: 0.1% TFA in acetonitrile.

All compounds are > 95% pure. The traces are included in Figures S1-S25.

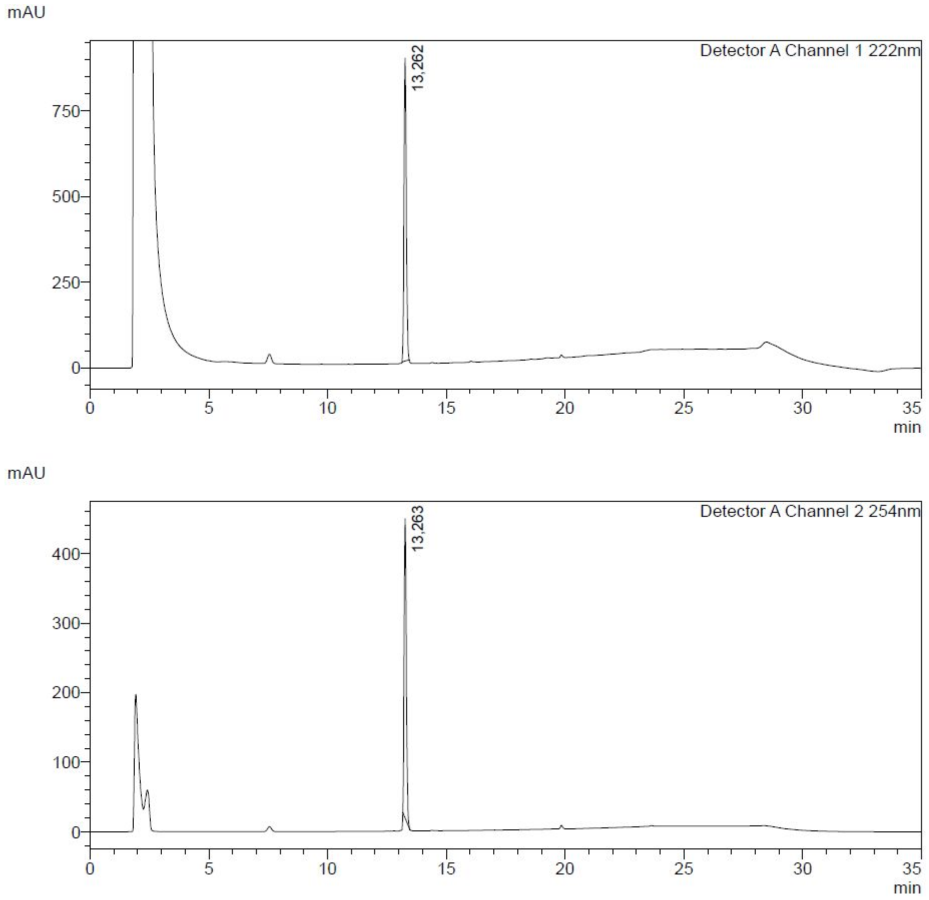

Figure S1. Analytical HPLC analysis of compound **30** (conditions **II**)

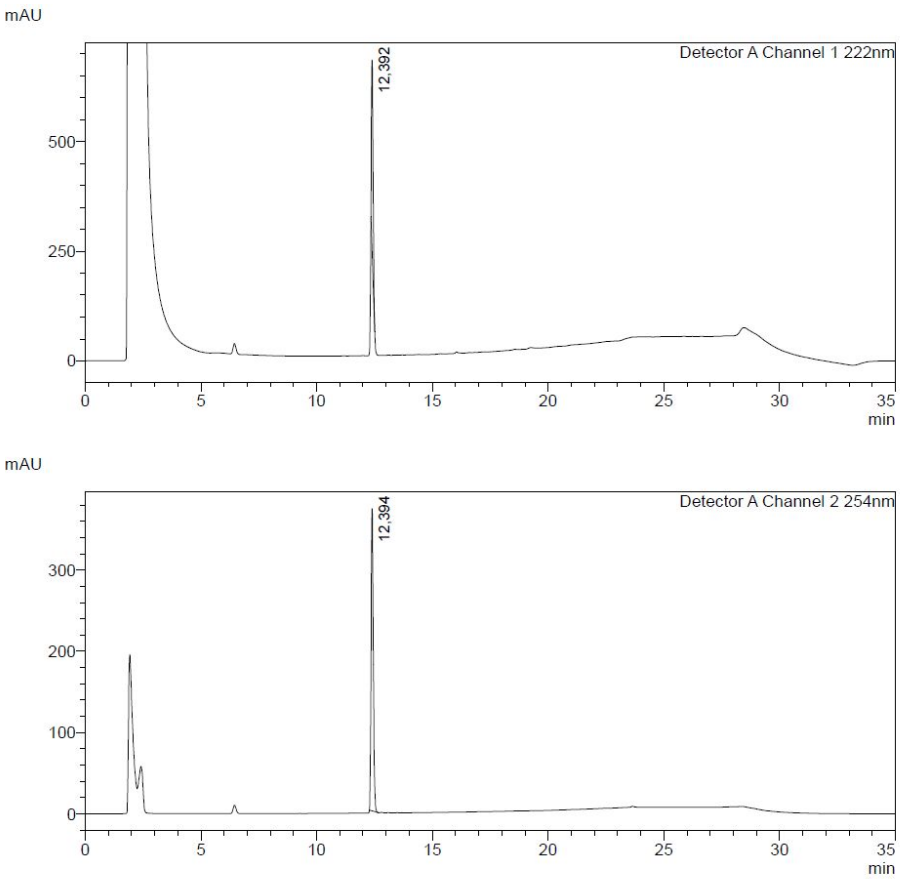

Figure S2. Analytical HPLC analysis of compound **31** (conditions **II**)

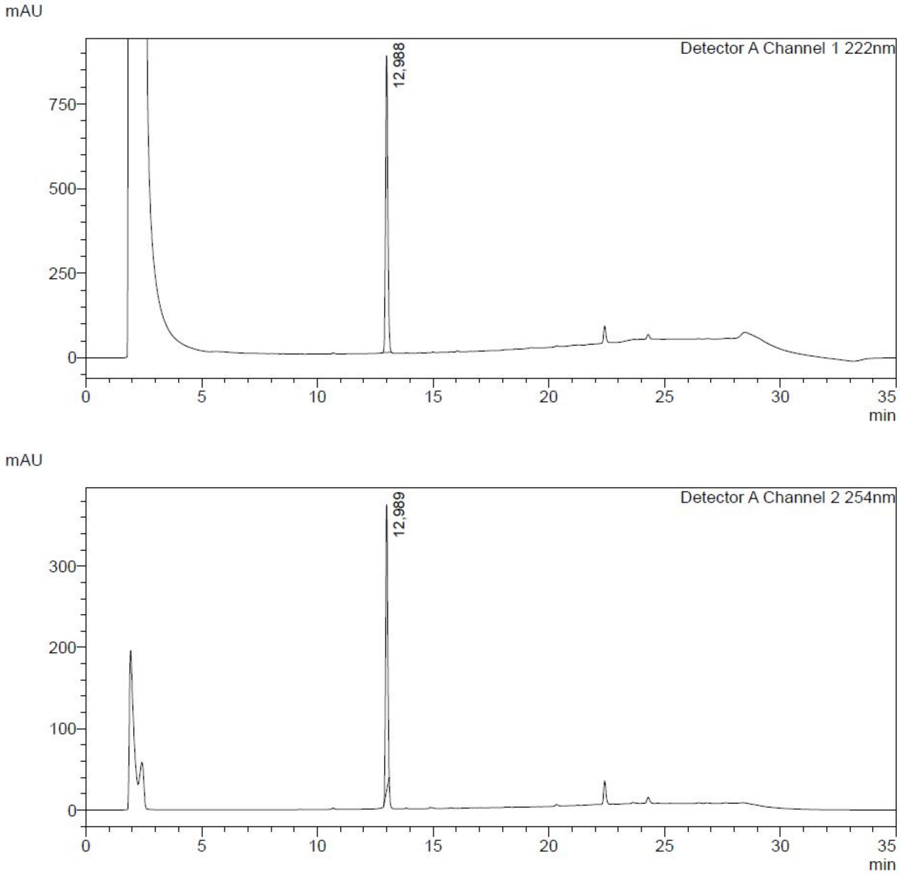

Figure S3. Analytical HPLC analysis of compound **32** (conditions **II**)

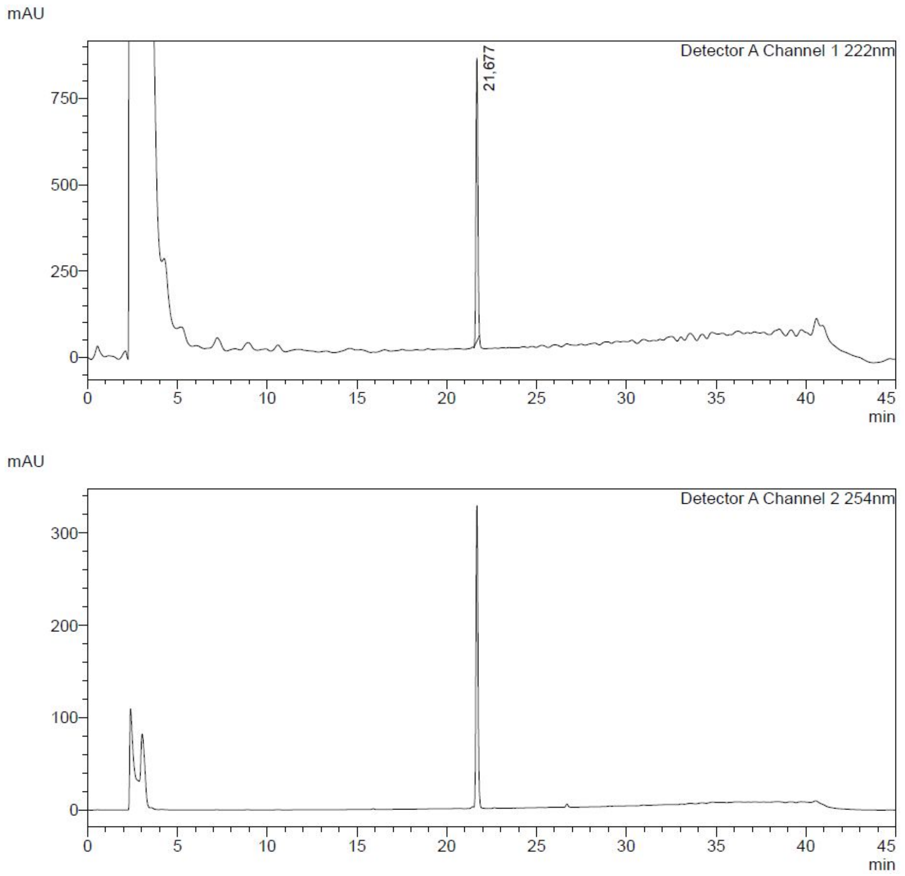

Figure S4. Analytical HPLC analysis of compound **33** (conditions **I**)

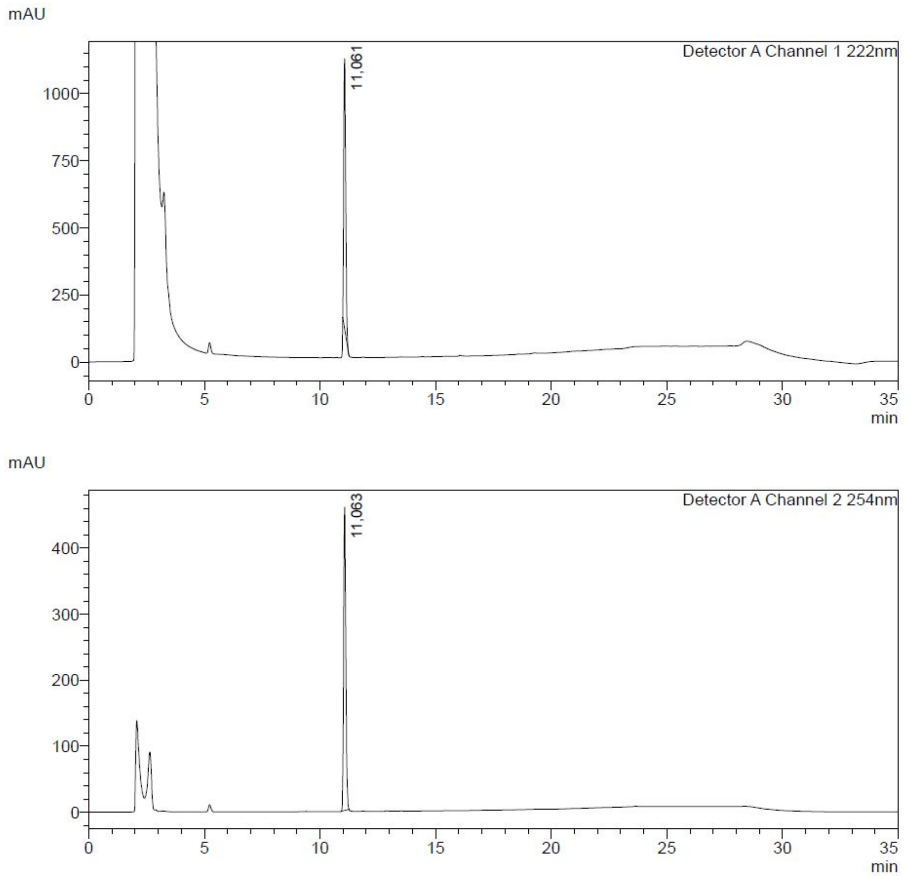

Figure S5. Analytical HPLC analysis of compound **34** (conditions **II**)

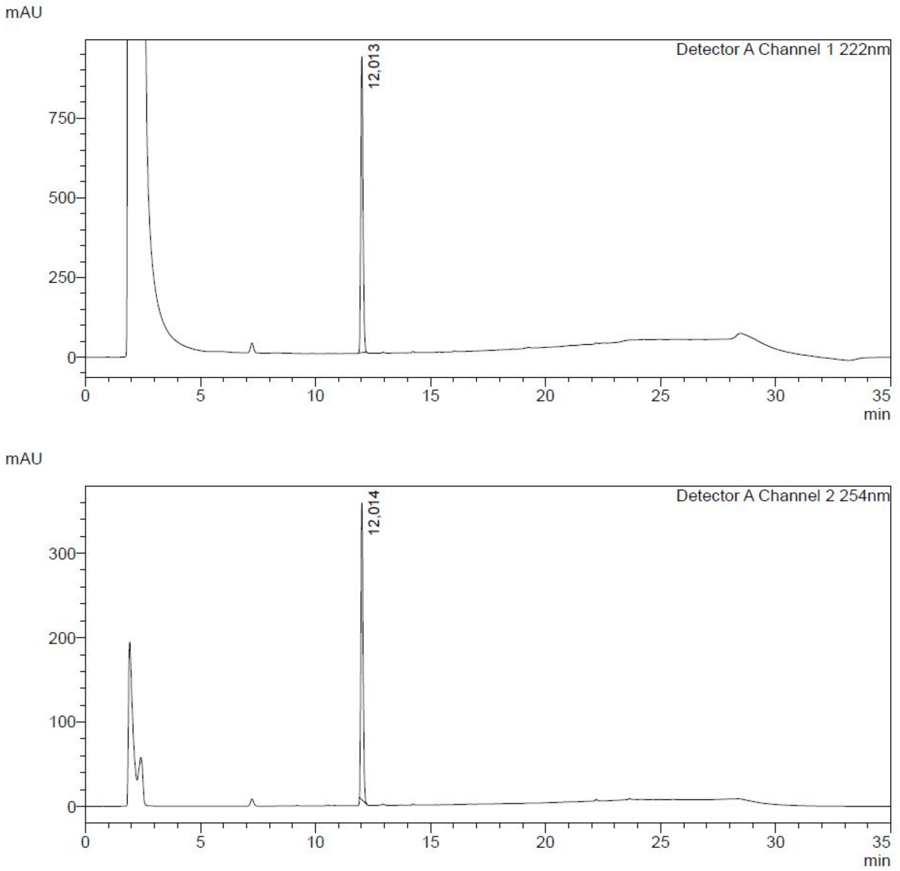

Figure S6. Analytical HPLC analysis of compound **35** (conditions **II**)

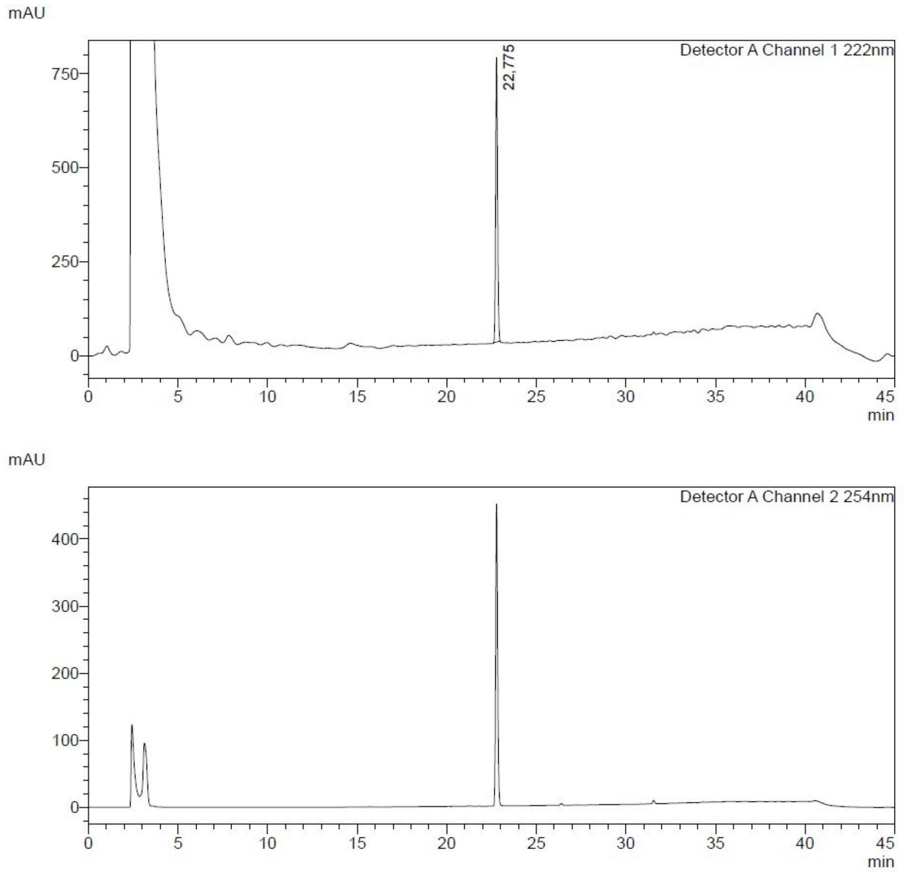

Figure S7. Analytical HPLC analysis of compound **36** (conditions **I**)

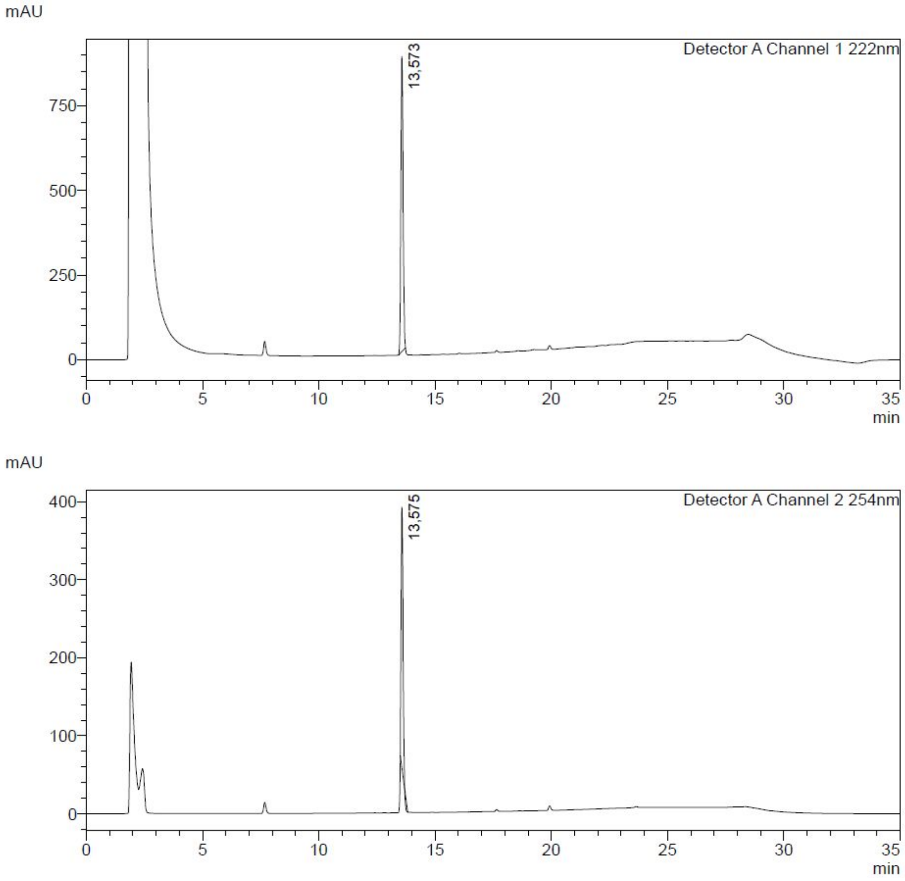

Figure S8. Analytical HPLC analysis of compound **37** (conditions **II**)

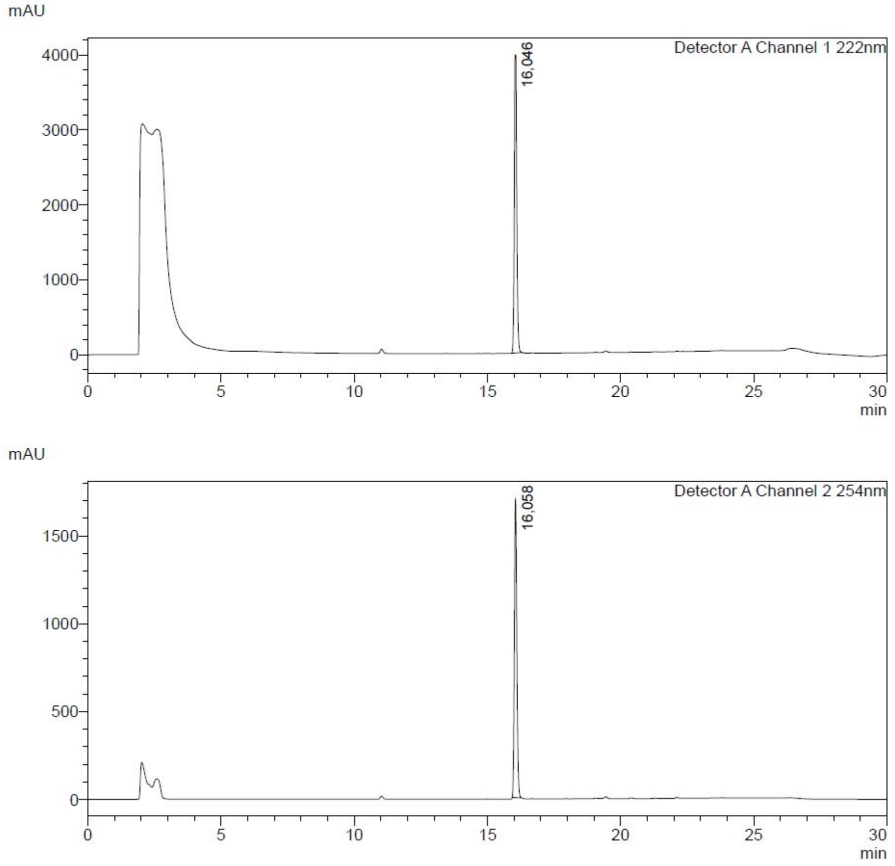

Figure S9. Analytical HPLC analysis of compound **38** (conditions **III**)

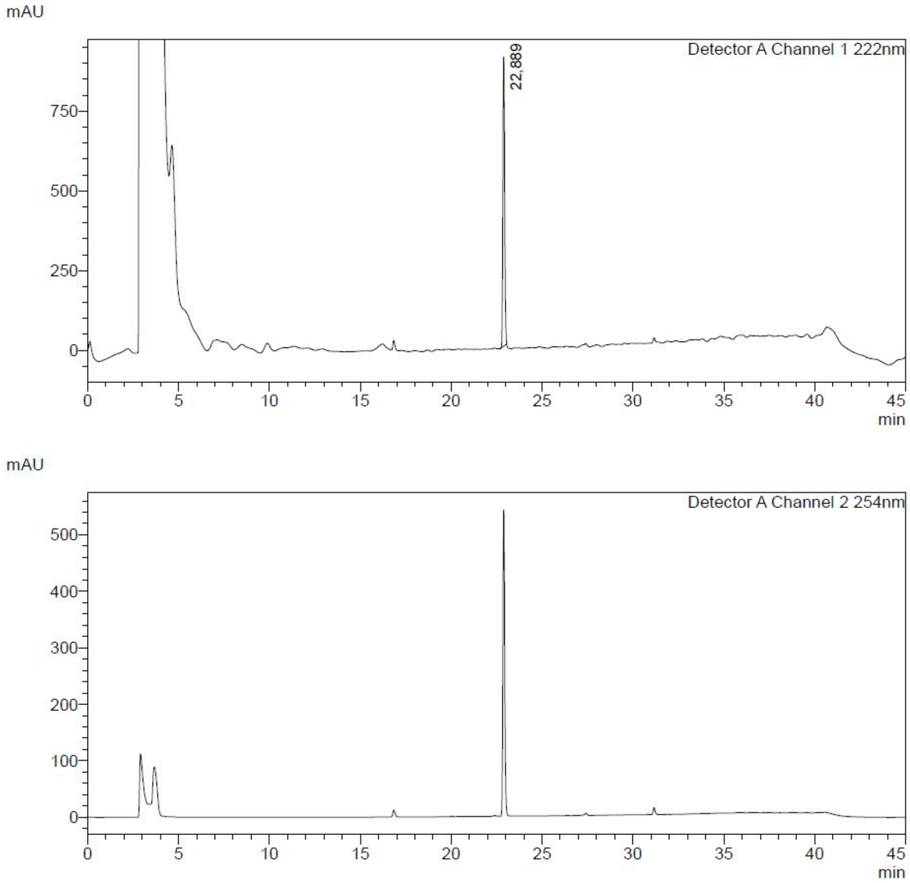

Figure S10. Analytical HPLC analysis of compound **39** (conditions **I**)

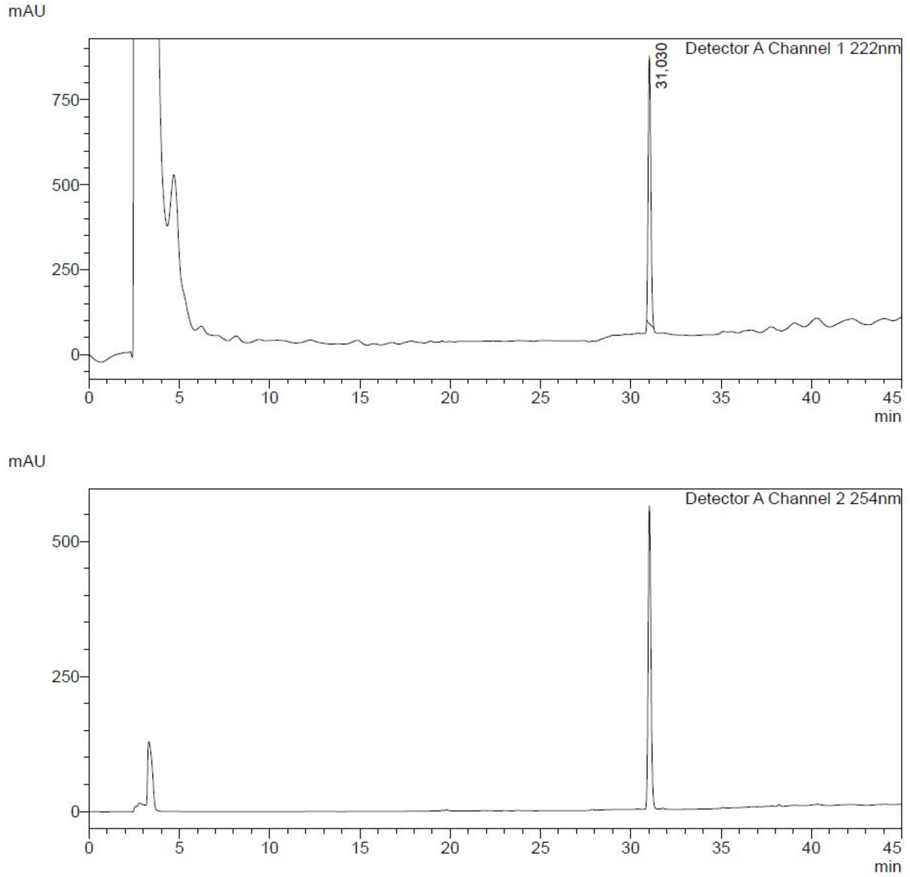

Figure S11. Analytical HPLC analysis of compound **40** (conditions **I**)

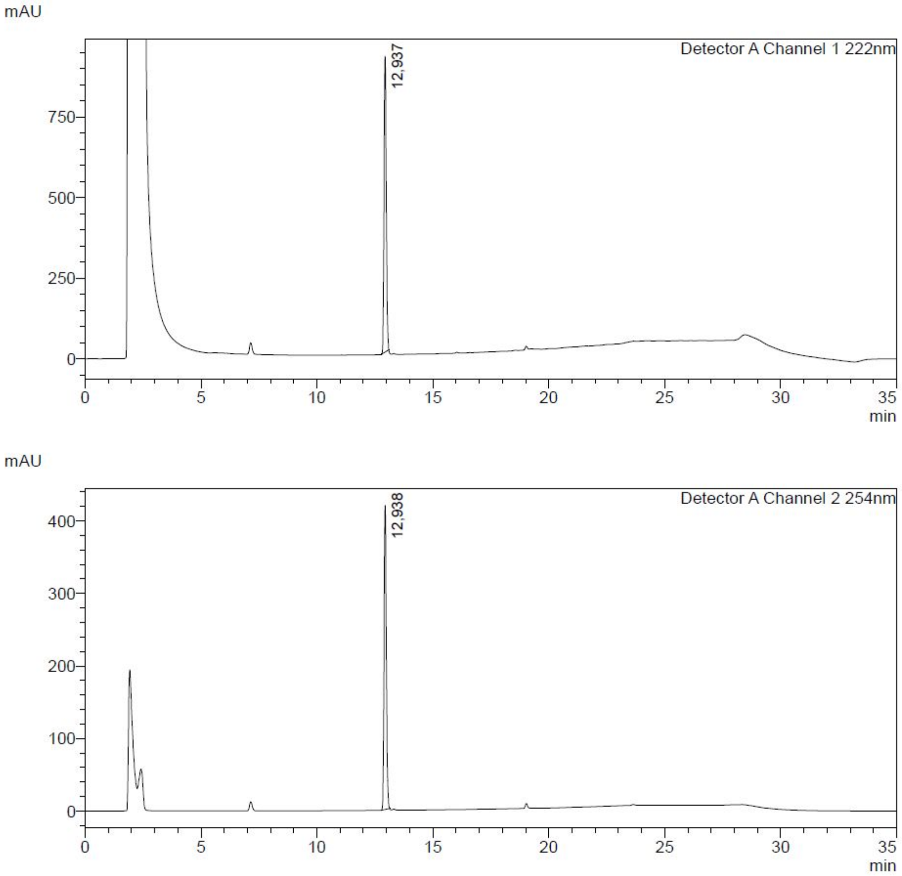

Figure S12. Analytical HPLC analysis of compound **41** (conditions **II**)

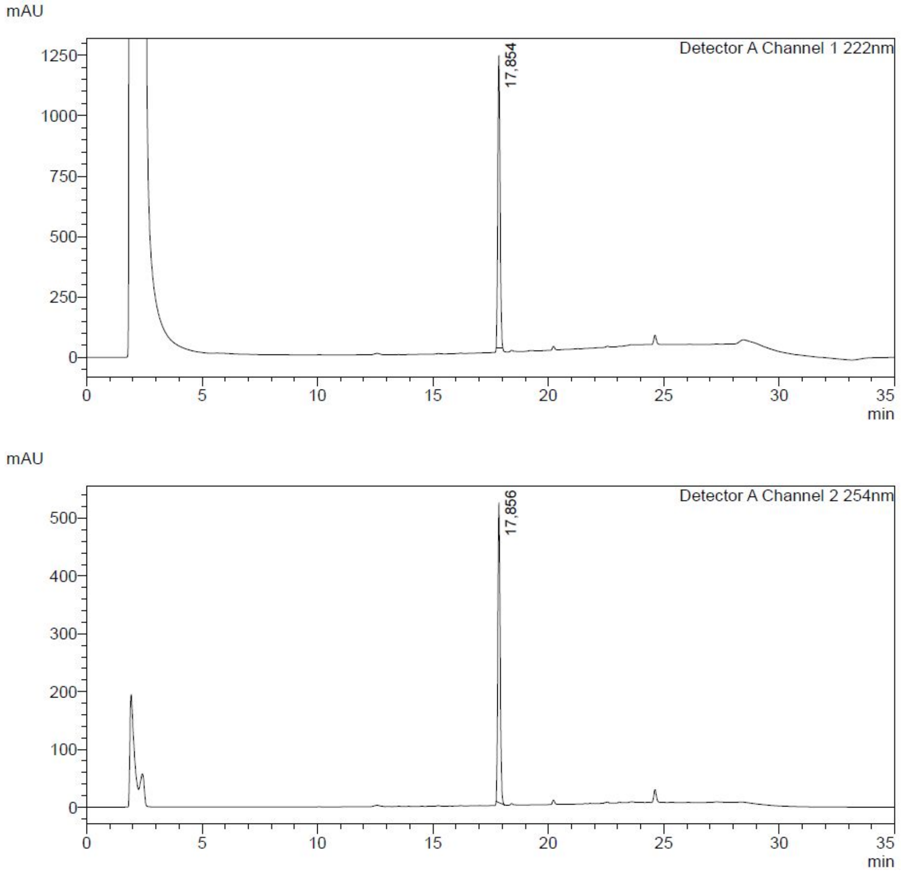

Figure S13. Analytical HPLC analysis of compound **42** (conditions **II**)

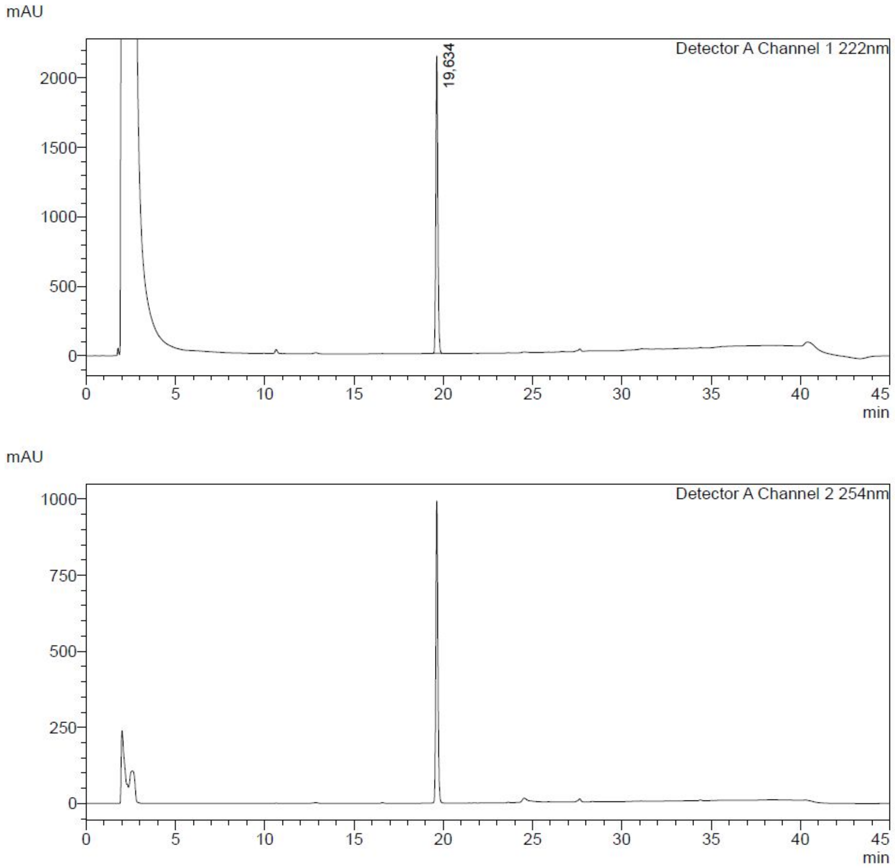

Figure S14. Analytical HPLC analysis of compound **43** (conditions **I**)

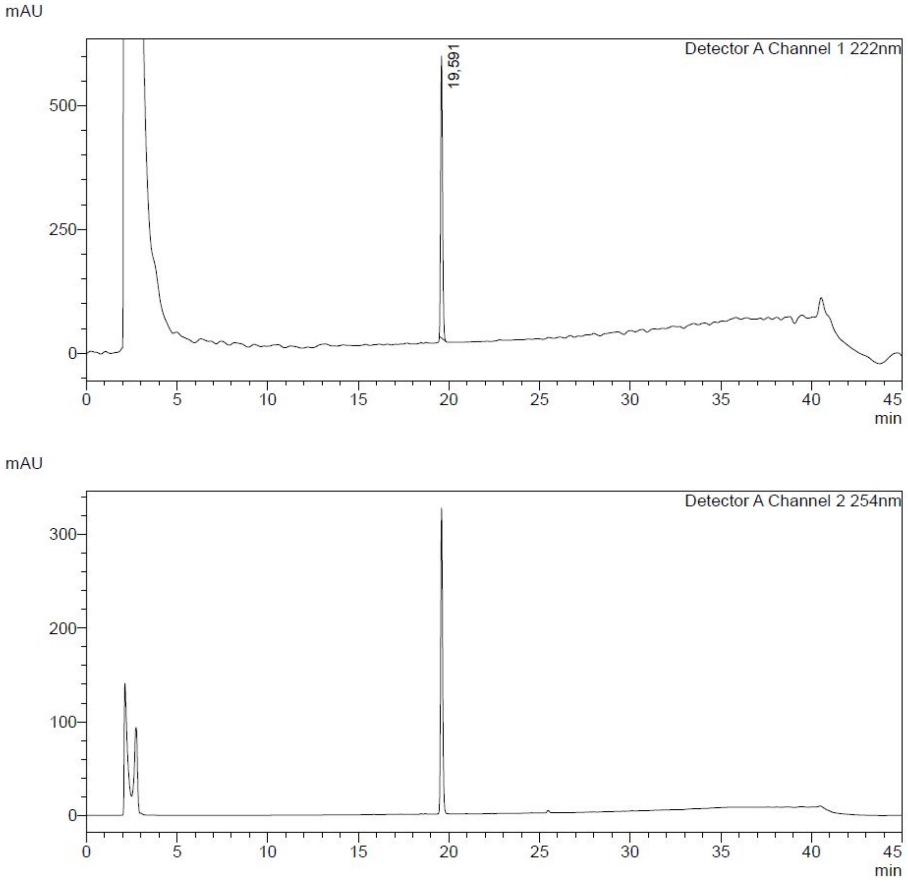

Figure S15. Analytical HPLC analysis of compound **44** (conditions **I**)

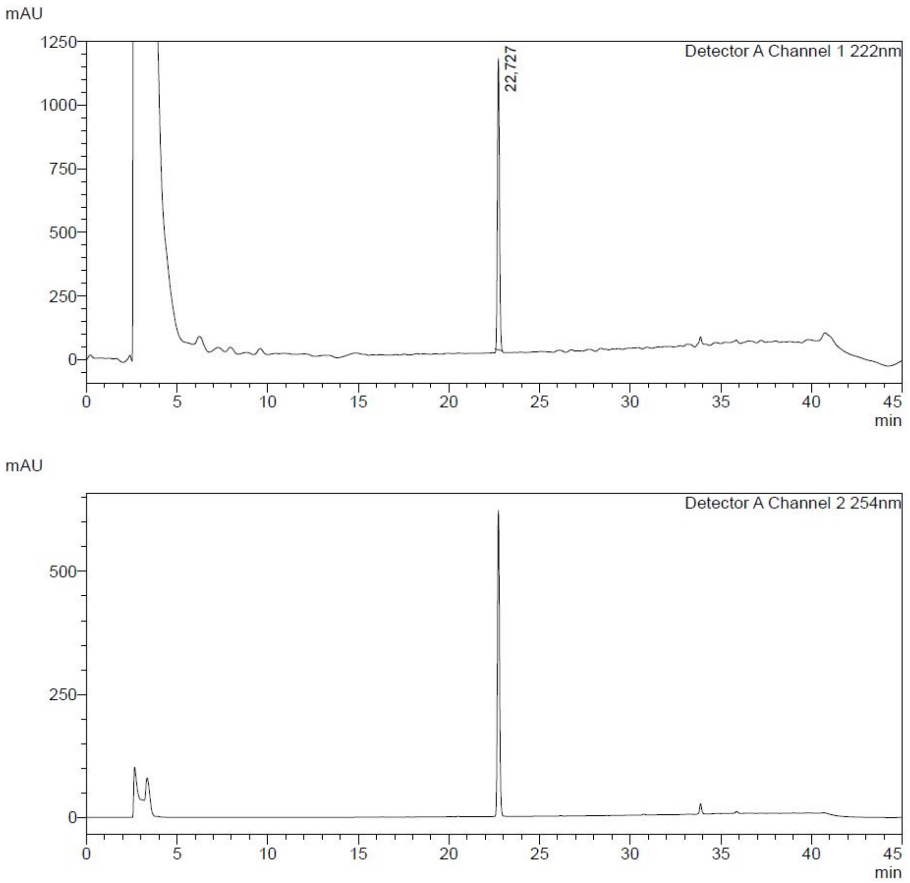

Figure S16. Analytical HPLC analysis of compound **45** (conditions **I**)

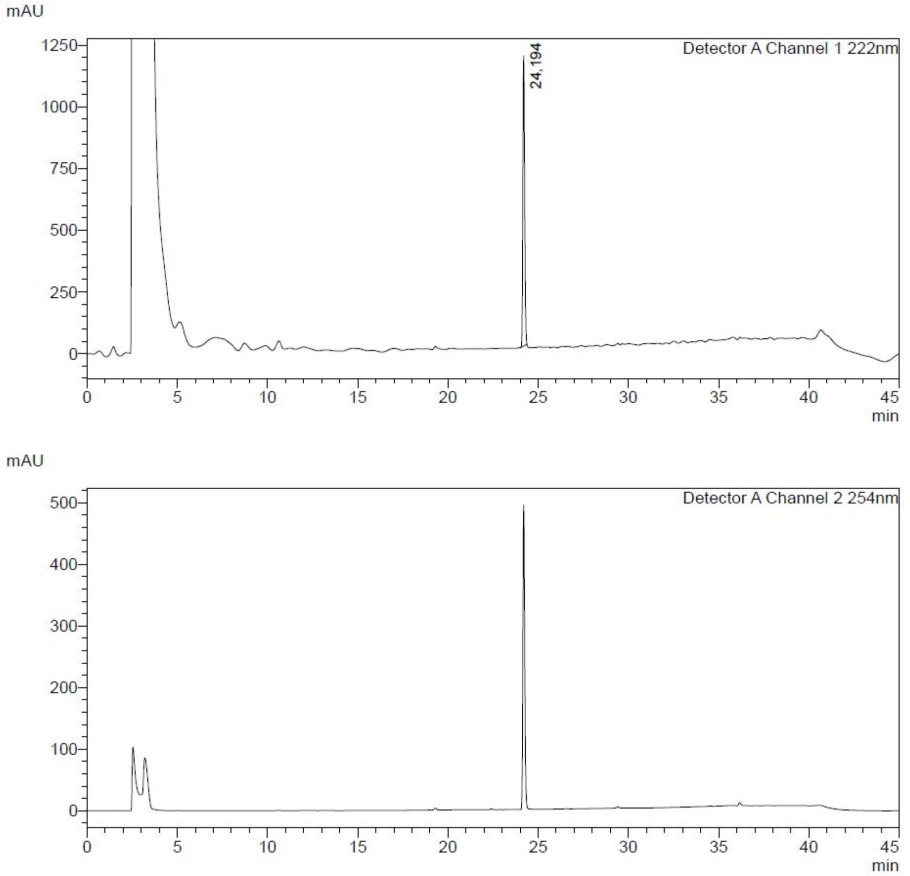

Figure S17. Analytical HPLC analysis of compound **46** (conditions **I**)

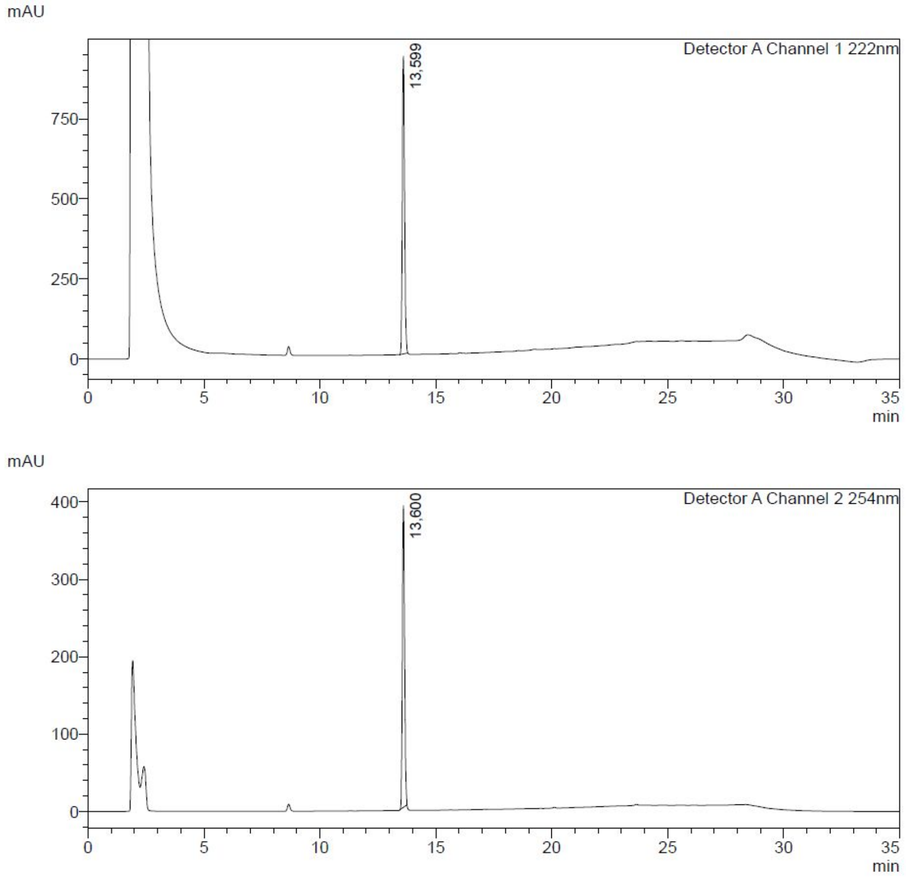

Figure S18. Analytical HPLC analysis of compound **47** (conditions **II**)

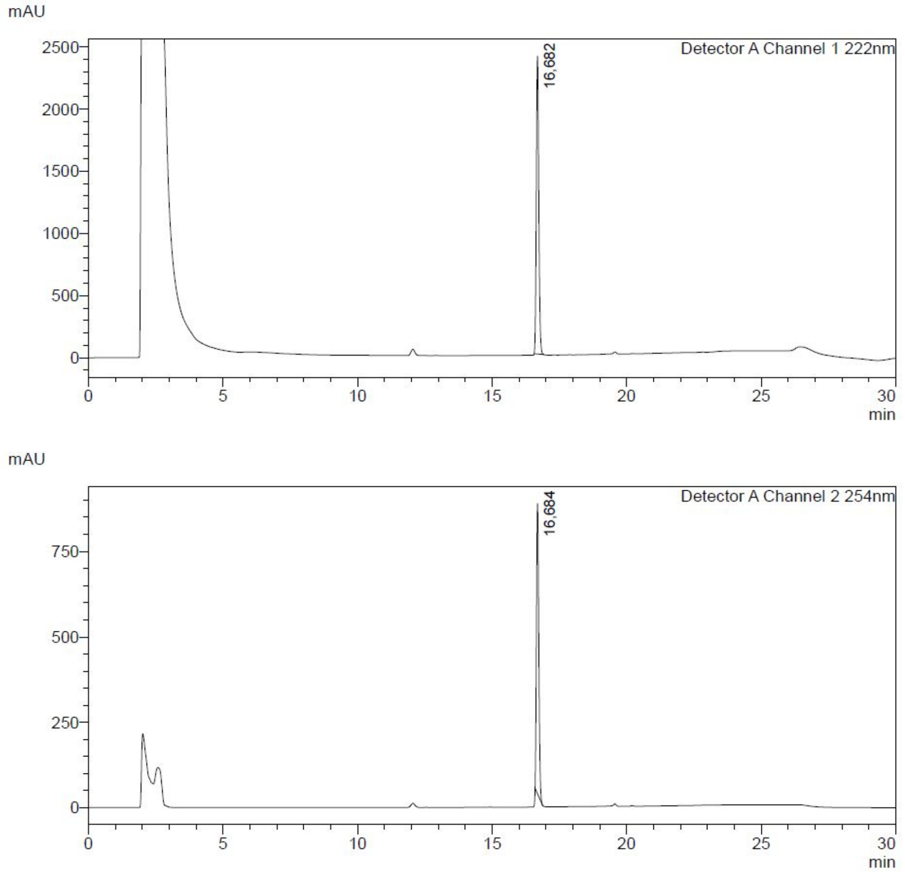

Figure S19. Analytical HPLC analysis of compound **48** (conditions **III**)

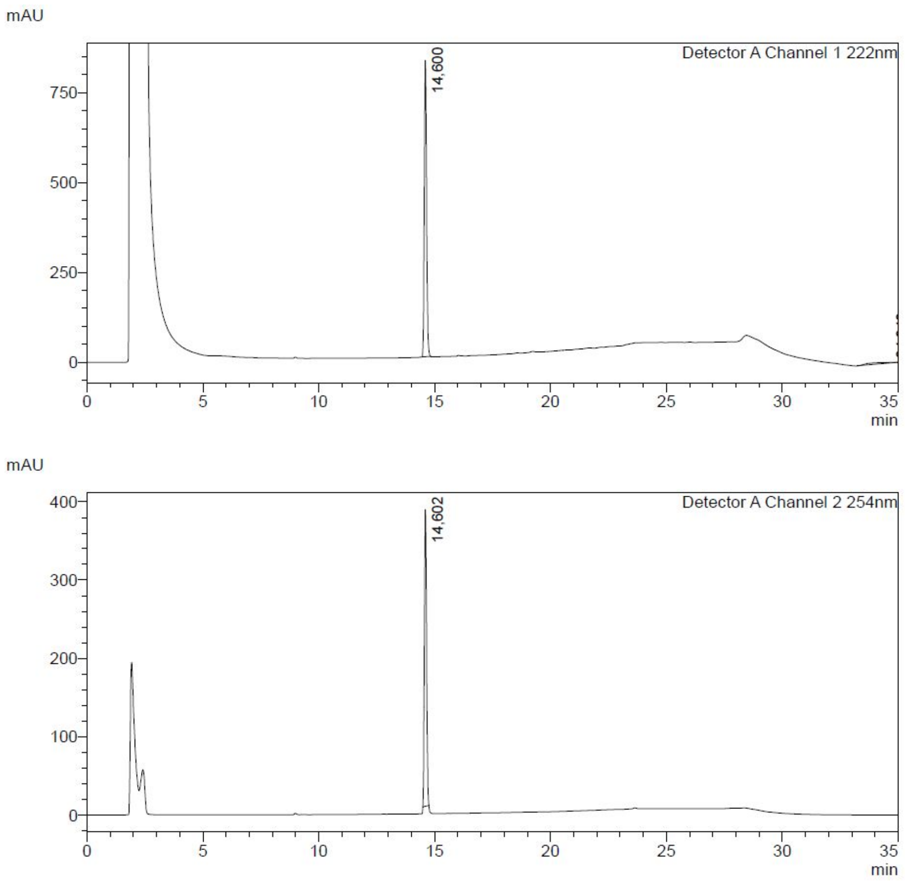

Figure S20. Analytical HPLC analysis of compound **49** (conditions **II**)

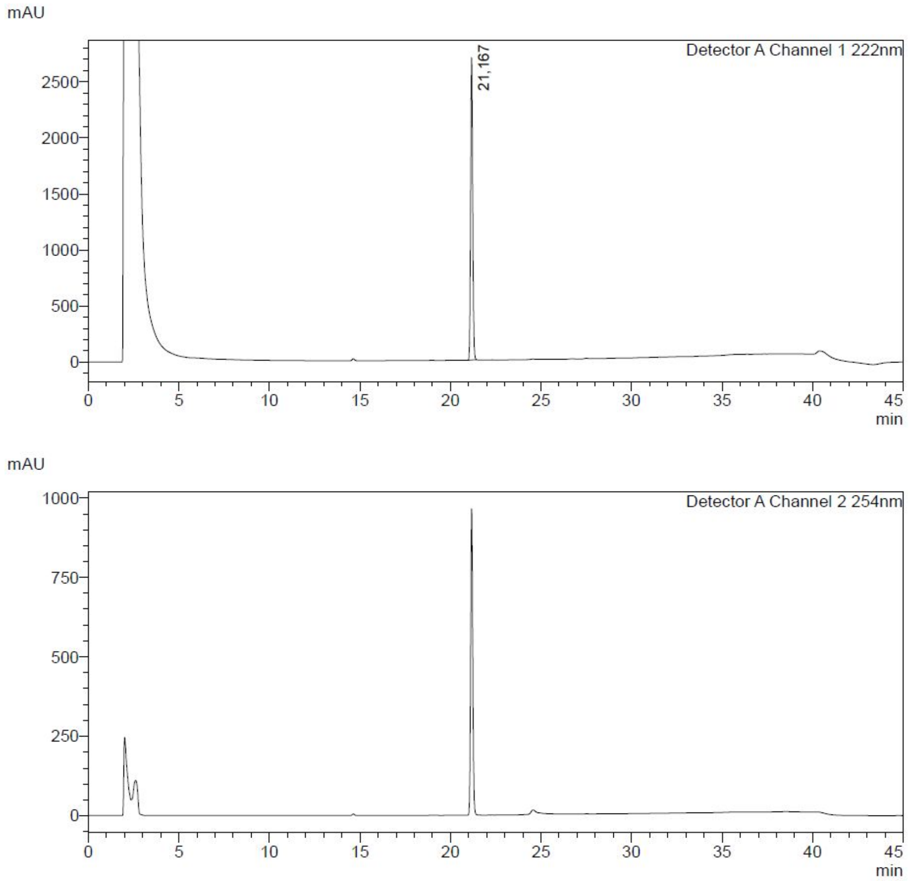

Figure S21. Analytical HPLC analysis of compound **50** (conditions **I**)

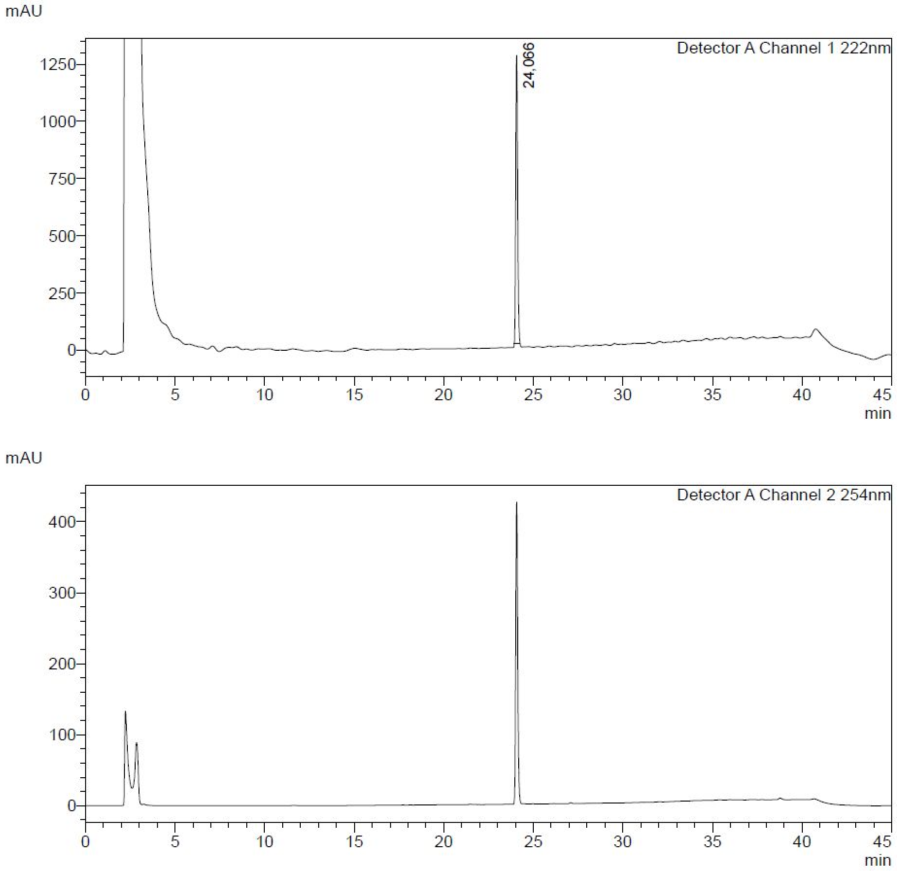

Figure S22. Analytical HPLC analysis of compound **51** (conditions **I**)

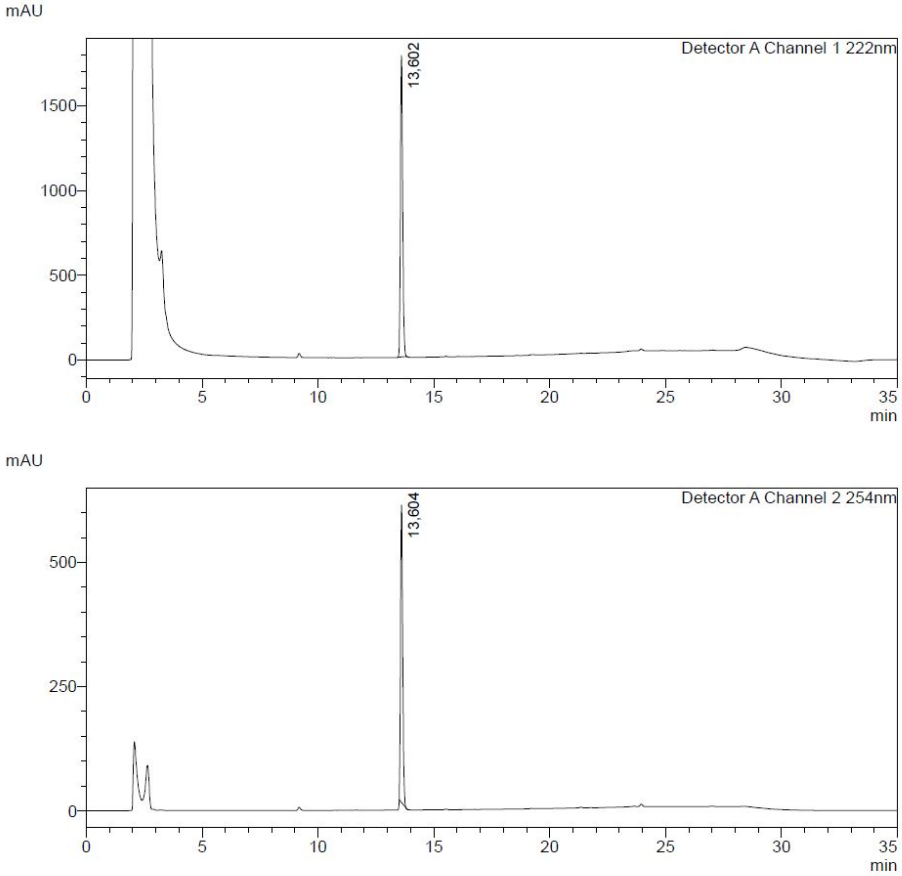

Figure S23. Analytical HPLC analysis of compound **52** (conditions **II**)

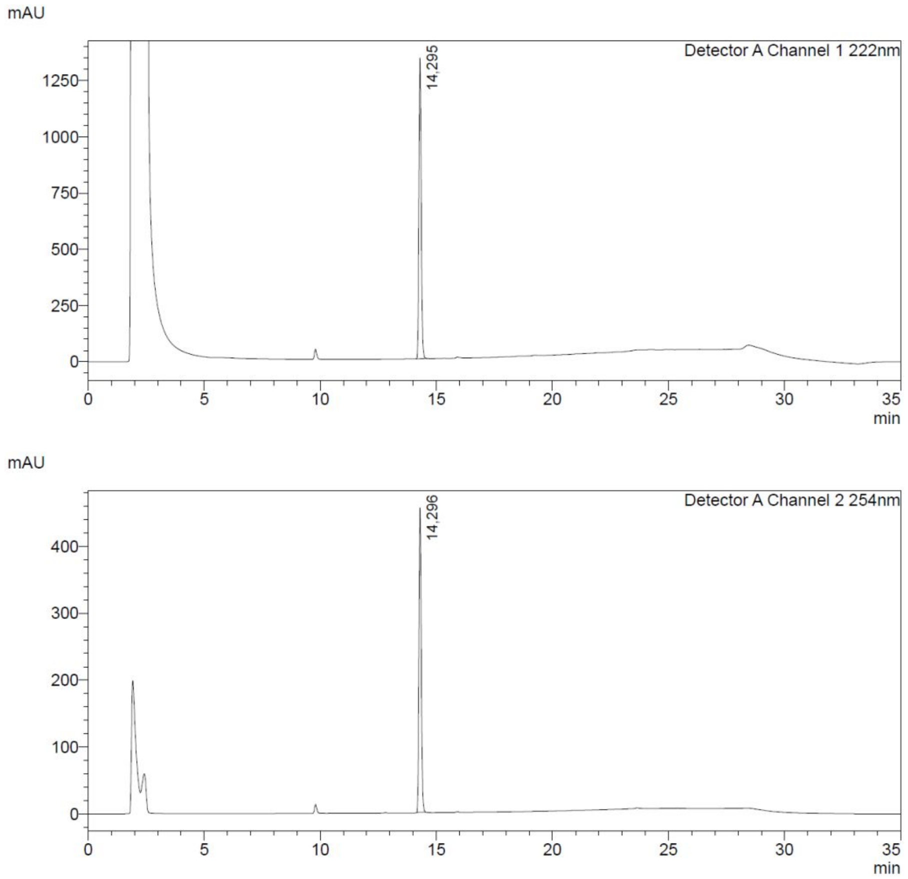

Figure S24. Analytical HPLC analysis of compound **53** (conditions **II**)

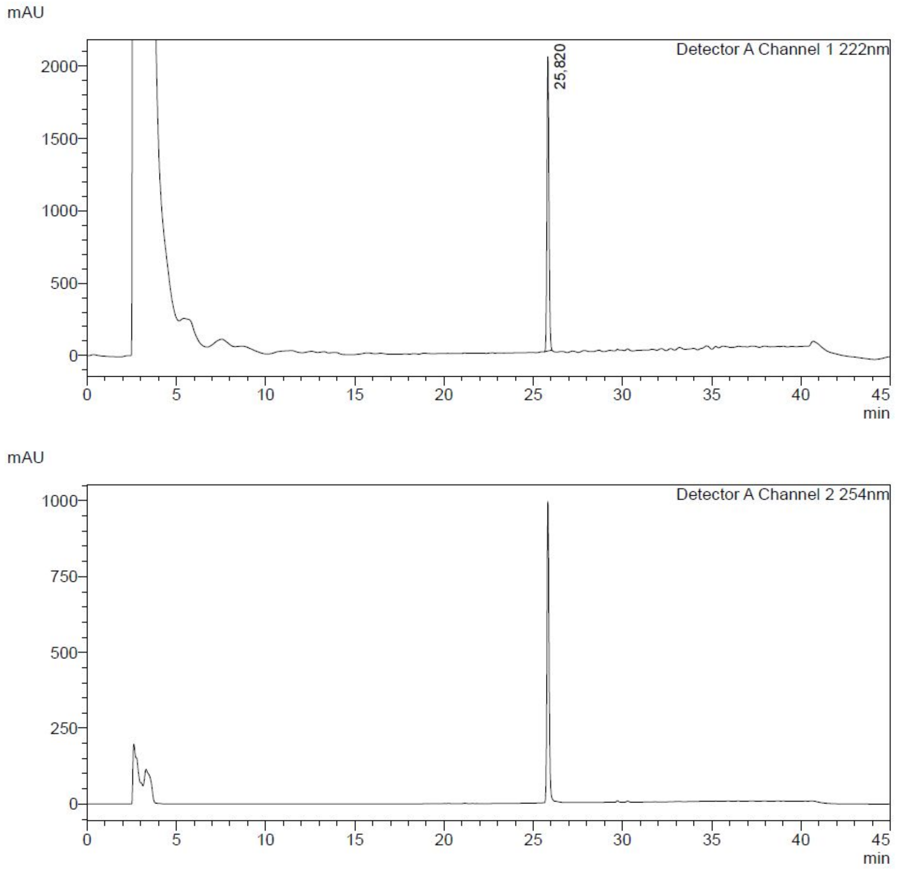

Figure S25. Analytical HPLC analysis of compound **54** (conditions **I**)

S2. Enzymatic studies. The native urease of *S. pasteurii* CCM 2056 was purified using a five-step chromatographic procedure, as previously described.[S10] The use of the multistep chromatographic approach allowed homogeneous preparation of urease. The values of kinetic parameters (K_M and v_{max}) of the purified enzyme were determined by fitting the initial reaction velocities measured in a range of urea concentrations to the Michaelis-Menten equation by nonlinear regression. The values obtained for *S. pasteurii* urease were K_M = 5.02 ± 0.28 mM, v_{max} = 4.109 ± 0.0588 μM s^{-1} with specific activity 2329 U/mg.

Enzymatic reactions were carried out in 3 mM phosphate buffer pH 7.5 at 37 °C. The kinetic parameters of urease in the noninhibited reaction (K_m and v_{max}) were obtained by measuring the initial rates of the reactions carried out in mixtures of urease from *S. pasteurii* (119.3 pM) and urea (1-60 mM) using the phenol-hypochlorite method, as previously reported.[S11] One unit (U) of enzyme activity was defined as the amount of enzyme required to produce 1 μM ammonia per min under specific conditions.

S2.1. Inhibition studies. Competitive reversible inhibition. Inhibition studies (compounds **30-36**, **38-41**, **44-51**, **53** and **54**) were carried out by initiating the enzyme reaction with the addition of 119.3 pM *S. pasteurii* urease in assay mixtures (200 μL total volume) containing increasing concentrations of inhibitors and 1−60 mM of urea. The K_i values were calculated using the appropriate equations in the GraphPad Prism 5 software. The inhibition mechanism was determined using Lineweaver-Burk plots after testing at least five inhibitor concentrations in the range depending on their inhibitory strength (exemplified in Figure S26).

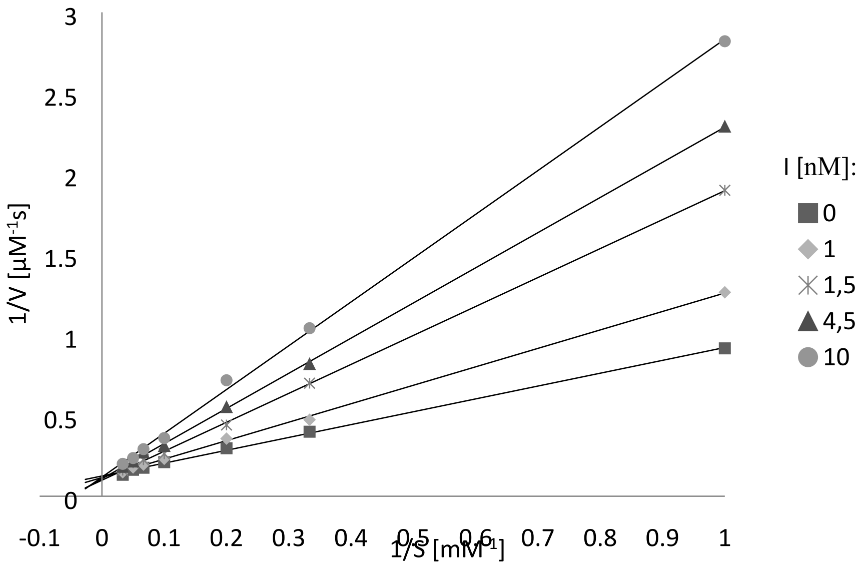

Figure S26. Lineweaver-Burk plot for inhibition of *S. pasteurii* urease by inhibitor **46**

S2.2. Inhibition studies. Slow binding inhibition. Reaction progress curves (compounds **37**, **42**, **43** and **52**), ammonia concentration versus time, for urea hydrolysis catalyzed by *S. pasteurii* urease in the absence and presence of inhibitors were performed in standard assay mixtures at a single urea concentration (final concentration 25 mM) and at different inhibitor concentrations according to their inhibitory strength. Enzymatic hydrolysis of urea was initiated by adding 500 µL of urease solution (final concentration 119.3 pM) to the reaction mixture (final volume 5 mL) and monitored for 120 min in samples taken from reaction mixtures at set time intervals. The reaction mixtures were stirred throughout the measurement period. The nonlinear reaction progress curves with time-dependent inhibitors of urease (for the nonincubated reaction and under conditions: $[I]_0 \gg [E]_0$) were described using the integrated equation: $[P] = v_s t + (v_i + v_s)(1 - e^{-k_{app}t})\frac{1}{k_{app}}$, (where P is the concentration of the reaction product formed at time t, which is the reaction time, v_i and v_s are the reaction initial and steady-state rates, respectively, and k_{app} is the apparent first-order rate constant for the interconversion between v_i and v_s). The control curves in the absence of inhibitor were linear. The equilibrium dissociation constants of the initial EI and the final enzyme conformation EI^* complex

(K_i and K_i^*, respectively) were determined using equation: $\frac{1}{v_{i(s)}} = \frac{K_M}{v_{max} S_0 K_i^{(*)}} I + \frac{1}{v_{max}}\left(1 + \frac{K_M}{S_0}\right)$ (where K_m is the Michaelis-Menten constant, v_{max} maximum rate of enzyme in a noninhibited reaction, S and I are substrate and inhibitor concentration). The mechanism of inhibitor binding was determined using steady-state kinetic measurements (Figures S27 and S28). The enzyme (final concentration 119.3 pM) was pre-incubated with increasing inhibitor concentrations in separate aliquots of reaction buffer prior to the initiation of the reaction by urea incorporation at a concentration ranging from 1 to 60 mM. The incubation time was different for the assayed inhibitors, depending on when equilibrium was achieved between enzyme (E), inhibitor (I) and enzyme-inhibitor complexes (EI and EI^*: $E + I \leftrightarrow EI \leftrightarrow EI^*$) . The K_i values were calculated using the appropriate equations implemented in the GraphPad Prism 5 software. The inhibitors were dissolved in water or DMF (the solvent was found to be non-inhibitory up to a concentration of 20%). Urease activity was determined using the Berthelot colorimetric reaction as previously described.[S11,S12] The determinations of K_i values were run in triplicate for the compounds analyzed.

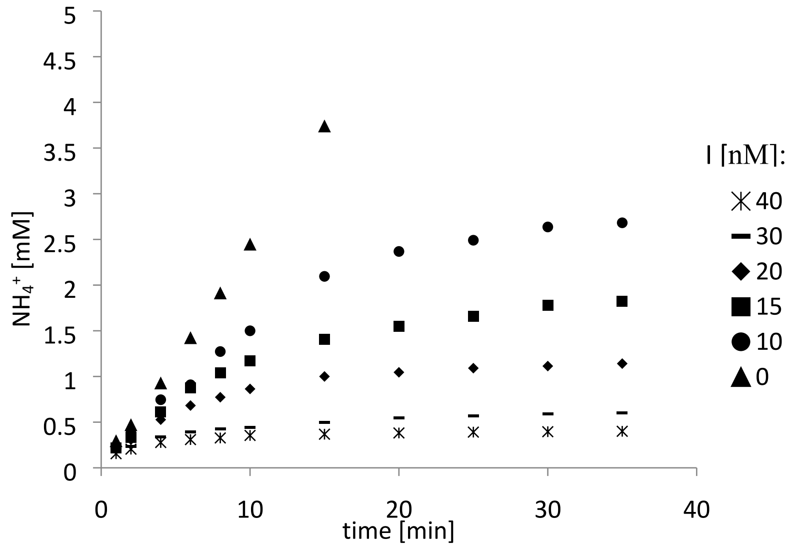

Figure S27. Progress curves for urea hydrolysis catalyzed by *S. pasteurii* urease in the presence of inhibitor **37**

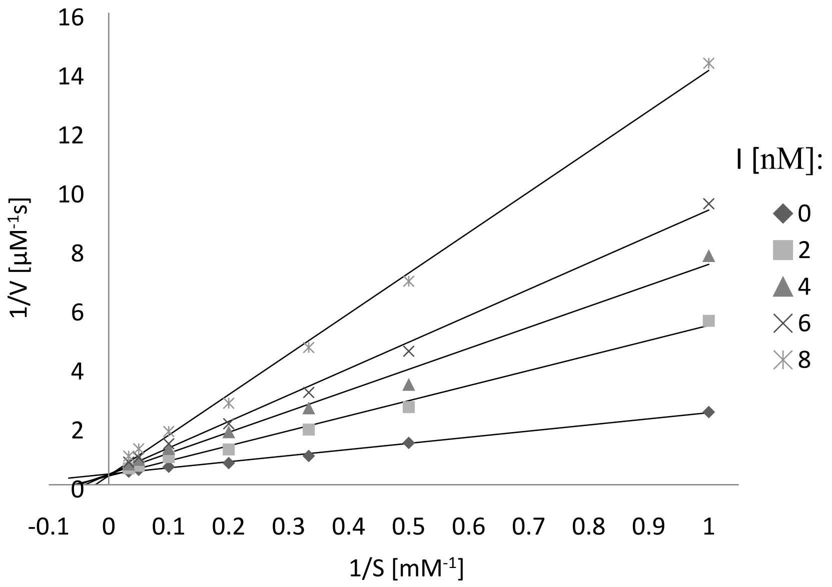

Figure S28. Lineweaver-Burk plot for inhibition of *S. pasteurii* urease by inhibitor **37**

S3. Inhibition of ureolysis in whole cells of *Proteus mirabilis*. *P. mirabilis* PCM543 was obtained from the Polish Collection of Microorganisms, Institute of Immunology and Experimental Therapy, Polish Academy of Sciences, Wroclaw, Poland. The strain was grown overnight in Mueller Hinton Broth 2 (Oxoid). The culture was then supplemented with sterile urea solution (2% final concentration) and nickel(II) chloride (200 µM final concentration). After 24 hours, cells were collected by centrifugation, washed with sterile 10 mM PBS and suspended in buffered peptone water to obtain OD_{650} of 0.5. Urease test was carried out on Thermo Scientific™ Nunc MicroWell 96-Well microplates, treated with non-tissue culture. Each well contained 100 µL assay mixture composed of 10 mM sterile PBS with 0.01% phenol red (w/v) and various concentrations of inhibitors tested. 10 µL aliquots of cell suspension were added and incubated in the presence of inhibitors at 25 °C for 1.5 hours, then sterile urea solution was added to obtain a concentration of 30 mM. The urease reaction was recorded at 25 °C as the absorbance change at 570 nm for 1.5 hours, using the TECAN Sunrise microplate reader. For the determination of inhibition of whole cell urease in urine model, modified artificial urine was used in which urea was omitted, sodium citrate was replaced with 0.5% glucose

and 0.01% phenol red added.[S13] The assays were carried out in tubes containing 2.0 mL of modified urine and various concentrations of compounds tested. Cells were induced to ureolysis and introduced into the reaction mixture in the proportion described above. After 1.5 hours of preincubation with gentle mixing at 25 °C, sterile urea solution (30 mM final concentration) was added and the urease reaction was carried out for another 1.5 hours. 120 µL samples were withdrawn every 10 min and briefly centrifuged to remove precipitate that can occur in artificial urine medium due to elevation of the pH induced by urease. The supernatant (100 µL) was placed in a microplate well and immediately read at 570 nm.

The IC_{50} and IC_{90} parameters expressed the specific inhibitor concentration at which the increase in absorbance per minute was 50% and 10% of the rate of the untreated control, respectively. The results were calculated from three independent assays using seven inhibitor concentrations.

***S3.1.* Proteus mirabilis *PCM543 viability control*.** The MTT [3-(4,5-dimethyl-2-thiazolyl)-2,5-diphenyl-2H-tetrazolium bromide] was obtained from Sigma-Aldrich. 10 µL of MTT solution (1 mg mL^{-1} in 10 mM PBS, pH 7.2) was added to 90 µL of urease reaction samples collected at the end of each assay, which contained cells exposed to the compounds tested for a total of 3 hours. After 1 hour of incubation at 25 °C, 100 µL of acidic isopropanol (1.5% (v/v) solution of hydrochloric acid in isopropanol) was added. MTT formazan was allowed to solubilize for 15 min and the absorbance was read at 550 nm.

S4. Molecular modeling. The crystal structure of *S. pasteurii* urease with 1.50 Å resolution (PDB id 5G4H) was used as the starting point for the calculations.[S14] The structure was prepared for the calculations using the Discovery Studio Visualizer v. 4.1 (BIOVIA): (a) hydrogen atoms were automatically added assuming a pH of 7.0, (b) the protonation of amino acid residues that form active sites was manually checked and adjusted, and (c) the partial charges of all atoms were assigned using the Momany-Rone algorithm. Minimization of the inhibitor-enzyme covalent complex (e.g. Figure

S29) was performed using the CHARMM program.[S15] Smart Minimizer algorithm and the CHARMM force field were used for this purpose. Minimization was performed up to an energy change of 0.0 or an RMS gradient of 0.1. Residues that did not form the active site cleft were frozen. No implicit solvent model was applied. The non-bond radius was set to 14 Å.

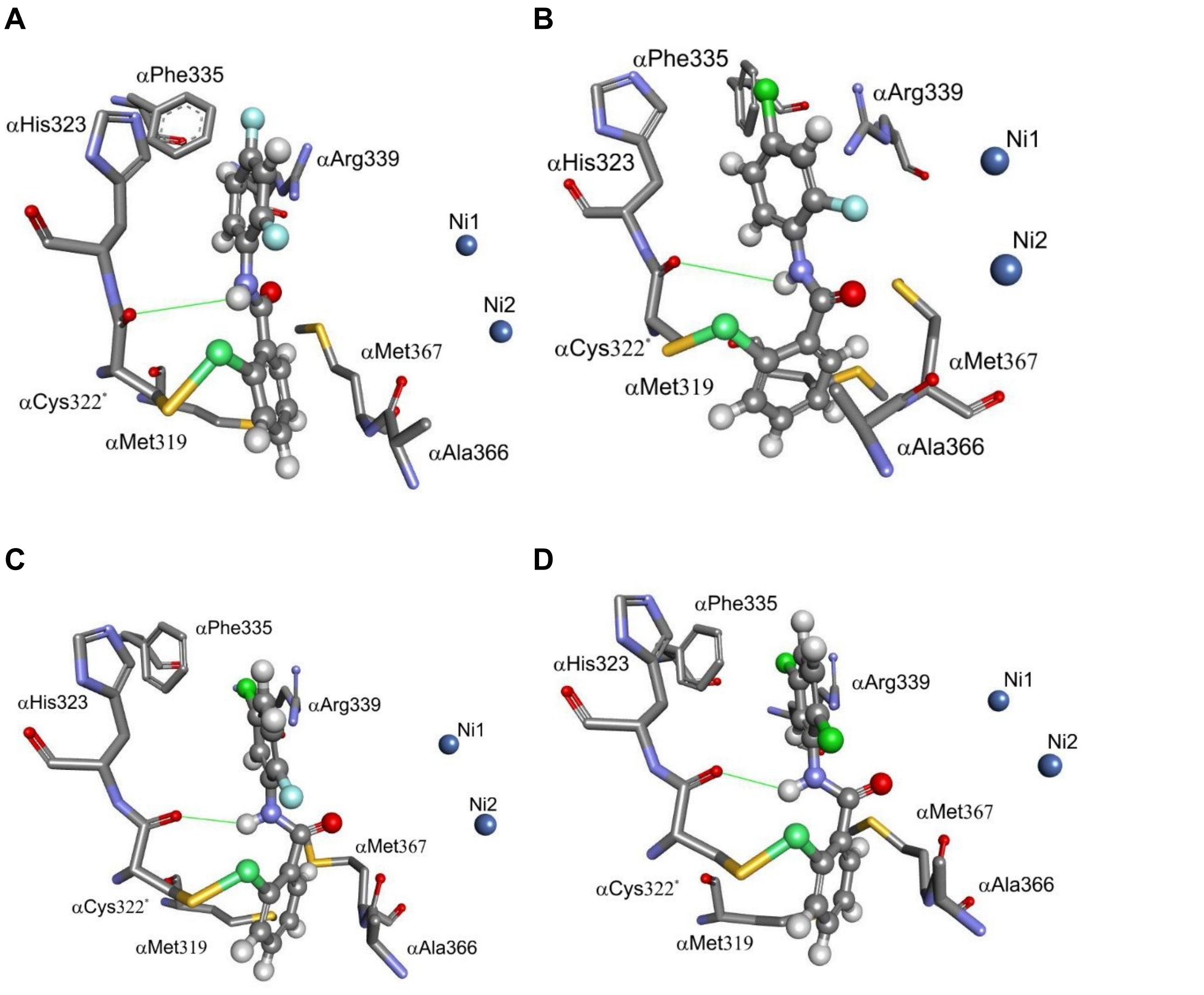

Figure S29. Modeled mode of binding of compounds **44** (A), **45** (B), **49** (C) and **51** (D) to the urease of *S. pasteurii*. Residues of the active site are shown as sticks, while the bound inhibitor is colored in ball and stick representation according to atom type (grey, carbon; blue, nitrogen; white, hydrogen; red, oxygen; orange, sulfur; light blue, fluorine; green, selenium; dark blue, nickel). The hydrogen bond is shown as a green thin solid line.

S5. Crystallization, data collection and structural determination. Incubation mixtures of *S. pasteurii* urease and **47** were carried out by adding a solution of 10 mM **47** dissolved in DMSO to an aliquot of 11 mg mL^{-1} *S. pasteurii* urease dissolved in 50 mM HEPES buffer at pH 7.5, at 1:10 dilution ratio (v/v), thus reaching a final **47** concentration of 1 mM. After an incubation time of 3 hours, necessary to abolish the enzyme activity, crystallization drops were deposited by diluting 1.5 µL of the *S. pasteurii* urease-**47** mixture with 1.5 µL of a precipitant solution consisting of 1.6-2.1 M $(NH_4)_2SO_4$ in 100 mM sodium citrate buffer, at pH 6.3. Crystallization trials were performed at 293 K using the vapor diffusion technique (hanging-drop method) using 24-well XRL Plates (*Molecular Dimensions*) and equilibrating the crystallization drops against 1 mL of the precipitant solution. Rice-shaped protein crystals appeared within approximately two weeks in the presence of 1.9-2.1 M $(NH_4)_2SO_4$ and grew up to 0.1 x 0.1 x 0.3 mm^3. Crystals were transferred in a solution containing 2.4 M $(NH_4)_2SO_4$, 100 mM sodium citrate buffer at pH 6.3, and 20% (v/v) ethylene glycol to ensure crystal cryoprotection, and then flash-cooled and stored in liquid nitrogen.

Diffraction data were collected at 100 K using synchrotron X-ray radiation at the EMBL P13 beamline of the Petra III storage ring, c/o DESY, Hamburg (Germany).[S16] Helical data collection was performed to achieve higher data quality by minimizing protein crystal radiation damage. Data processing and reduction were carried out using XDS[S17] and AIMLESS.[S18,S19] The crystals belonged to space group $P6_322$, isomorphous with all the crystal structures of *S. pasteurii* urease determined so far. The initial phases for the structure determination were obtained using the X-ray crystal structure of *S. pasteurii* urease bound to catechol (PDB code 5G4H, 1.50 Å resolution)[S14] as a phasing model, devoid of solvent molecules, catechol and other ligands, and following coordinates randomization in order to remove any potential phase bias. Structural determination was conducted by restrained refinement with REFMAC5.[S20] Model rebuilding, as well as water or ligand addition/inspection, were manually conducted using COOT.[S21,S22] Unbiased omit electron density maps for non-proteinaceous ligands were calculated using Fourier coefficients F_o - F_c and phases from the last cycle of restrained refinement before the addition of the ligands in the refining model.

The X-ray crystal structure was refined using isotropic atomic displacement parameters (ADPs) (including the hydrogen atoms in the riding positions), at a final resolution of 1.54 Å, and deposited in the Protein Data Bank with the accession code 7ZCY. Data collection, processing and final refinement statistics are given in Table S1. Selected distances and angles around the Ni(II) ions in the present structure were compared with the corresponding data obtained for the crystal structure of native *S. pasteurii* urease (PDB code 4CEU, Table S2). The active site and mobile flap regions of both structures are superimposed in Figure S30.

Figures were generated using PyMol (The PyMOL Molecular Graphics System, v. 2.0 Schrödinger, LLC.).

S5.1. Quantum mechanical calculations. The energetics and structure of the neutral Me-S-Se-SeH and the anionic Me-S-Se-Se$^-$ moieties were evaluated using quantum mechanical calculations. The structure of both moieties was built using Spartan'20 v. 1.1.4 (Wavefunction Inc.), energy minimized using simple molecular mechanics, followed by energy minimization using density functional at the wB97X-D/6-311+G(2df,2p) level. Selected distances and angles around the Se atoms in the crystal structure of *S. pasteurii* urease bound to Se after crystallization in the presence of **47** were compared with the same parameters obtained from quantum mechanics calculations (Table S3).

Table S1. Data collection, processing and refinement statistics (PDB code 7ZCY)

**Data collection and processing**	
Wavelength (Å)	0.9763
Detector	DECTRIS PILATUS 6M
Crystal-to-Detector distance (mm)	270.686
Oscillation angle (degrees)	0.1
Number of images	1800
Space group	$P6_322$
Unit cell (*a, b, c,* Å)	131.61, 131.61, 189.00
Resolution range (Å)[a]	189.00-1.54 (1.57-1.54)
Total number of reflections[a]	2775124 (139107)
Unique reflections[a]	142031 (6976)
Multiplicity[a]	19.5 (19.9)
Completeness[a] (%)	100.0 (100.0)
R_{sym}[a,b] (%)	9.4 (230.1)
R_{pim}[a,c] (%)	3.1 (75.2)
Mean I half-set correlation CC(1/2)[a]	1.000 (0.749)
Mean I/σ(I)[a]	20.9 (1.6)
**Refinement statistics**	
Number of monomers in the asymmetric unit	3
R_{factor}[d] (%)	13.7
R_{free}[d] (%)	16.0
Cruickshank's DPI for coordinate error[e] based on R_{factor} (Å)	0.056
Wilson plot B-factor (Å^2)	21.2
Average all atom B-factor[f] (Å^2)	27.5
B-factor for the Ni atoms (Å^2)	21.9; 21.1
B-factor for the Se atoms (Å^2)	82.4; 90.7
RMS (bonds)[d]	0.013
RMS (angles)[d]	1.81
Total number of atoms	7302
Total number of water molecules	787
Solvent content (%)	54.93
Matthews Coefficient (Å^3/Da)	2.73
**Ramachandran plot**	
Most favored regions (%)	89.2
Additionally allowed regions (%)	9.8
Generously allowed regions (%)	0.8
Disallowed regions (%)	0.2

[a] Highest resolution bin in parentheses;

[b] $R_{sym} = \sum_{hkl}\sum_j |I_j - \langle I \rangle| / \sum_{hkl}\sum_j I_j$, where I is the intensity of a reflection, and ⟨I⟩ is the mean intensity of all symmetry related reflections j; [c]$R_{p.i.m.} = \sum_{hkl}\{[1/(N-1)]^{1/2}\sum_j |I_j - \langle I \rangle|\}/\sum_{hkl}\sum_j I_j$, where I is the intensity of a reflection, and ⟨I⟩ is the mean intensity of all symmetry related reflections j, and N is the multiplicity;

[d]Taken from REFMAC;[S20] R_{free} is calculated using 5% of the total reflections that were randomly selected and excluded from refinement;

[e]$DPI = R_{factor} \cdot D_{max} \cdot compl^{-1/3}\sqrt{\frac{N_{atoms}}{(N_{refl} - N_{params})}}$, where N_{atoms} is the number of the atoms included in the refinement, N_{refl} is the number of the reflections included in the refinement, D_{max} is the maximum resolution of reflections included in the refinement, *compl* is the completeness of the observed data, and for isotropic refinement, $N_{params} \approx 4N_{atoms}$.[S23]

Table S2. Selected distances (Å) and angles (°) around the Ni(II) ions in the crystal structure of *S. pasteurii* urease bound to Se after crystallization in the presence of **47**. The same distances belonging to the crystal structure of native *S. pasteurii* urease (PDB code 4CEU), are reported as a comparison.

**PDB code** **Ni - L Distances (Å)**	**7ZCY @ 1.54 Å**	**4CEU @ 1.58 Å**
Ni(1) - αLys220* Oθ1	2.0	1.9
Ni(1) - O_B[a]	2.1	2.1
Ni(1) - O_1	2.1	2.2
Ni(1) - αHis249 Nδ	2.0	2.0
Ni(1) - αHis275 Nε	2.0	2.0
Ni(2) - αLys220* Oθ2	2.1	2.1
Ni(2) - O_B	2.1	2.1
Ni(2) - O_2	2.1	2.1
Ni(2) - αHis137 Nε	2.1	2.1
Ni(2) - αHis139 Nε	2.0	2.1
Ni(2) - αAsp363 Oδ1	2.1	2.1
Ni(1) ••• Ni(2)	3.7	3.7
**L - Ni - L Angles (°)**		
αLys220* Oθ1 - Ni(1) - αHis249 Nδ	101.8	100.4
αLys220* Oθ1 - Ni(1) - αHis275 Nε	105.0	107.2
αLys220* Oθ1 - Ni(1) - O_B	94.1	96.6
αLys220* Oθ1 - Ni(1) - O_1	107.8	108.2
αHis249 Nδ - Ni(1) - αHis275 Nε	97.3	98.6
αHis275 Nε - Ni(1) - O_B	99.2	94.6
O_B - Ni(1) - O_1	63.3	67.0
O_1 - Ni(1) - αHis249 Nδ	91.1	89.3
αHis249 Nδ - Ni(1) - O_B	153.3	154.2
αHis275 Nε - Ni(1) - O_1	143.7	141.6
αLys220* Oθ2 - Ni(2) - αHis137 Nε	93.9	90.8
αLys220* Oθ2 - Ni(2) - αHis139 Nε	91.4	91.7
αLys220* Oθ2 - Ni(2) - O_2	91.2	92.9
αLys220* Oθ2 - Ni(2) - O_B	94.1	95.6
αAsp363 Oδ1 - Ni(2) - αHis137 Nε	81.8	82.8
αAsp363 Oδ1 - Ni(2) - αHis139 Nε	86.2	86.4
αAsp363 Oδ1 - Ni(2) - O_2	94.2	94.5
αAsp363 Oδ1 - Ni(2) - O_B	90.5	89.1
O_2 - Ni(2) - O_B	64.5	67.7
O_B - Ni(2) - αHis137 Nε	95.6	95.0
αHis137 Nε- Ni(2) - αHis139 Nε	109.3	108.5
αHis139 Nε- Ni(2) - O_2	89.7	88.4
αLys220* Oθ2 - Ni(2) - αAsp363 Oδ1	174.0	172.4
O_B - Ni(2) - αHis139 Nε	153.7	155.3
O_2- Ni(2) - αHis137 Nε	160.1	162.6
Ni(1) - O_B - Ni(2)	124.6	122.1

[a] O_B, O_1 and O_2 indicate the water/hydroxide molecules in the bridging position and bound to Ni(1) and Ni(2), respectively.

Table S3. Selected distances (Å) and angles (°) around the two Se atoms in the crystal structure of *S. pasteurii* urease bound to Se after crystallization in the presence of **47**. A comparison with the same parameters obtained from quantum mechanics DFT calculations is also provided.

	**X-ray**	**DFT Me-S-Se-Se-H**	**DFT Me-S-Se-Se^{-}**
Se(1) – αCys322* Sγ	2.3	2.183	2.234
Se(1) – Se(2)	2.4	2.347	2.332
Se(2) – Se(1) – αCys322* Sγ	114.1	105.01	106.33
Se(1) – αCys322* Sγ – αCys322* Cβ	111.5	102.81	100.90
Se(2) – Se(1) – αCys322* Sγ – αCys322* Cβ	98.7	82.21	66.07

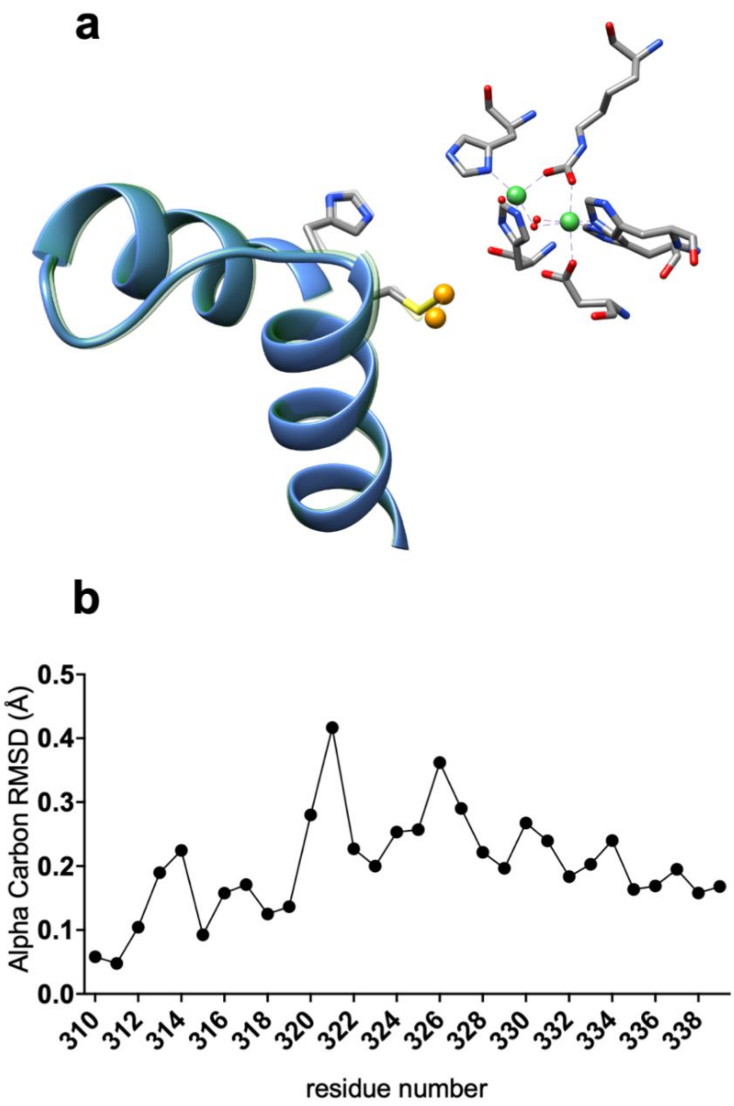

Figure S30. (a) Close-up of mobile flap and active site regions of *S. pasteurii* urease bound to the di-nuclear Se cluster, superimposed to the same environment of the crystal structure of the native enzyme (PDB code 4CEU).[S24] The mobile flaps are represented as ribbons and colored light blue and transparent green for the Se-bound and the native structures, respectively. Side chains of the selected residues are shown as stick, where carbon, nitrogen, oxygen, and sulfur atoms are grey, blue, red and yellow, respectively (transparent representation for those belonging to the native enzyme). The Ni and Se atoms are shown as green and orange spheres, respectively. (b) Plot showing the *per* residue Cα RMSD of the mobile flap region of the determined crystal structure calculated with respect to the crystal structure of native *S. pasteurii* urease (PDB code 4CEU).

S7. References

[S1] Piętka-Ottlik, M.; Burda-Grabowska, M.; Woźna, M.; Waleńska, J.; Kaleta, R.; Zaczyńska, E.; Piasecki, E.; Giurg M. Synthesis of new alkylated and methoxylated analogues of ebselen with antiviral and antimicrobial properties. *Arkivoc* **2017**, 546–556, and the references cited therein.

[S2] Chang, T.-C.; Huang, M.-L.; Hsu, W.-L.; Hwang, J.-M.; Hsu, L.-Y. Synthesis and biological evaluation of ebselen and its acyclic derivatives. *Chem. Pharm. Bull.* **2003**, **51**, 1413–1416.

[S3] Balkrishna, S. J.; Bhakuni, B. S.; Kumar, S. Copper catalyzed/mediated synthetic methodology for ebselen and related isoselenazolones. *Tetrahedron* **2011**, *67*, 9565–9575.

[S4] Węglarz-Tomczak, E.; Burda-Grabowska, M.; Giurg, M.; Mucha A. Identification of methionine aminopeptidase 2 as a molecular target of the organoselenium drug ebselen and its derivatives/analogues: synthesis, inhibitory activity and molecular modeling study. *Bioorg. Med. Chem. Lett.* **2016**, *26*, 5254–5259.

[S5] Giurg, M.; Gołąb, A.; Suchodolski, J.; Kaleta, R.; Krasowska, A.; Piasecki, E.; Piętka-Ottlik, M. Reaction of bis[(2-chlorocarbonyl)phenyl] diselenide with phenols, aminophenols, and other amines towards diphenyl diselenides with antimicrobial and antiviral properties. *Molecules* **2017**, *22*, 974.

[S6] Zmudzinski, M.; Rut, W.; Olech, K.; Granda, J.; Giurg, M.; Burda-Grabowska, M.; Zhang, L.; Sun, X.; Lv, Z.; Nayak, D.; Kesik-Brodacka, M.; Olsen, S.; Hilgenfeld, R.; Drag, M. Ebselen derivatives are very potent dual inhibitors of SARS-CoV-2 proteases - PL^{pro} and M^{pro} in in vitro studies. *bioRxiv - Biochemistry* Pub. Date: 2020-08-31, DOI: 10.1101/2020.08.30.273979.

[S7] Weglarz-Tomczak, E.; Tomczak, J. M.; Talma, M.; Burda-Grabowska, M.; Giurg, M.; Brul, S. Identification of ebselen and its analogues as potent covalent inhibitors of papain-like protease from SARS-CoV-2. *Sci. Rep.* **2021**, *11*, 3640.

[S8] Qiao, Z.; Wei, N.; Jin, L.; Zhang, H.; Luo, J.; Zhang, Y.; Wang, K. The Mpro structure-based modifications of ebselen derivatives for improved antiviral activity against SARS-CoV-2 virus. *Bioorg Chem.* **2021**, *117*, 105455.

[S9] Huff, S.; Kummetha, I. R.; Tiwari, S. K.; Huante, M. B.; Clark, A. E.; Wang, S.; Bray, W.; Smith, D.; Carlin, A. F.; Endsley, M.; Rana, T. M. Discovery and mechanisms of SARS-CoV-2 main protease inhibitors. *J. Med. Chem.* **2022**, *65*, 2866–2879.

[S10] Macegoniuk, K.; Dziełak, A.; Mucha, A.; Berlicki, Ł. Bis(aminomethyl)phosphinic acid, a highly promising scaffold for the development of bacterial urease inhibitors. *ACS Med. Chem. Lett.* **2015**, *6*, 146–150.

[S11] Vassiliou, S.; Grabowiecka, A.; Kosikowska, P.; Yiotakis, A.; Kafarski, P.; Berlicki, Ł. Design, synthesis and evaluation of novel organophosphorus inhibitors of bacterial ureases. *J. Med. Chem.* **2008**, *51*, 5736–5744.

[S12] Weatherburn, M. W. Phenol-hypochlorite reaction for determination of ammonia. *Anal. Chem.* **1967**, *39*, 971–974.

[S13] Brooks, T.; Keevil, C. W. A simple artificial urine for the growth of urinary pathogens. *Lett. Appl. Microbiol.* **1997**, *24*, 203–206.

[S14] Mazzei, L.; Cianci, M.; Musiani, F.; Lente, G.; Palombo, M.; Ciurli, S. Inactivation of urease by catechol: Kinetics and structure. *J. Inorg. Biochem.* **2017**, *166*, 182–189.

[S15] Brooks, B. R.; Brooks III, C. L.; Mackerell, A. D.; Nilsson, L.; Petrella, R. J.; Roux, B.; Won, Y.; Archontis, G.; Bartels, C.; Boresch, S.; Caflisch, A.; Caves, L.; Cui, Q.; Dinner, A. R.; Feig, M.; Fischer, S.; Gao, J.; Hodoscek, M.; Im, W.; Kuczera, K.; Lazaridis, T.; Ma, J;. Ovchinnikov, V.; Paci, E.; Pastor, R. W.; Post, C. B.; Pu, J. Z.; Schaefer, M.; Tidor, B.; Venable, R. M.; Woodcock, H. L.; Wu, X.; Yang, W.; York, D. M.; Karplus, M. CHARMM: The biomolecular simulation program. *J. Comp. Chem.* **2009**, *30*, 1545–1615.

[S16] Cianci, M.; Bourenkov, G.; Pompidor, G.; Karpics, I.; Kallio, J.; Bento, I.; Roessle, M.; Cipriani, F.; Fiedler, S.; Schneider, T. R. P13, the EMBL macromolecular crystallography beamline at the low-emittance PETRA III ring for high- and low-energy phasing with variable beam focusing. *J. Synchrotron Rad.* **2017**, *24*, 323–332.

[S17] Kabsch, W. XDS. *Acta Cryst.* **2010**, *D66*, 125–132.

[S18] Evans, P. Scaling and assessment of data quality. *Acta Cryst.* **2006**, *D62*, 72–82.

[S19] Evans, P. R. An introduction to data reduction: space-group determination, scaling and intensity statistics. *Acta Cryst.* **2011**, *D67*, 282–292.

[S20] Murshudov, G. N.; Vagin, A. A.; Dodson, E. J. Refinement of macromolecular structures by the maximum-likelihood method. *Acta Cryst.* **1997**, *D53*, 240–255.

[S21] Emsley, P.; Cowtan, K. *Coot*: model-building tools for molecular graphics. *Acta Cryst.* **2004**, *D60*, 2126–2132.

[S22] Emsley, P. Lohkamp, B. Scott, W. G.; Cowtan, K. Features and development of *Coot*. *Acta Cryst.* **2010**, *D66*, 486–501.

[S23] Cruickshank, D. W. Remarks about protein structure precision. *Acta Cryst.* **1999**, *D55*, 583–601.

[S24] Benini, S.; Cianci, M.; Mazzei, L.; Ciurli, S. Fluoride inhibition of *Sporosarcina pasteurii* urease: Structure and thermodynamics. *J. Biol. Inorg. Chem.* **2014**, *19*, 1243–1261.
